# Matrix‐Activated Aptamer Sensors for Human Serum Analysis

**DOI:** 10.1002/anie.3409471

**Published:** 2026-09-02

**Authors:** Amandine Barbaroux, Benoit Chovelon, Emmanuelle Fiore, Sebastien Vezirian, Mickael Henry, Marwa Zoubeidi, Maria C. DeRosa, Corinne Ravelet, Eric Peyrin

**Affiliations:** ^1^ DPM UMR 5063 University Grenoble Alpes ‐ CNRS Grenoble France; ^2^ Department of Biochemistry, Toxicology and Pharmacology Grenoble Site Nord CHU – Biology and Pathology Institute Grenoble France; ^3^ Department of Chemistry Carleton University Ottawa Canada

**Keywords:** aptamer, fluorescent sensor, human serum, matrix proteins, small‐molecule

## Abstract

Owing to their ease of operation, solution‐phase fluorescent sensors hold a crucial position among aptamer‐based detection tools. However, their performance is regularly compromised when exposed to increasing levels of blood fluids, such as human serum, which limits their potential in clinical settings. Here, we introduce a sensing platform that behaves advantageously within this biological medium, leveraging the most abundant proteins in the matrix, namely albumin and immunoglobulin G, as endogenous activators of fluorophore‐labeled aptamers. In the absence of a target, these serum proteins non‐specifically assemble with the aptamer probe in situ. Upon target binding, the aptamer‐protein complexes are disrupted, shifting the equilibria toward the probe unbound to biopolymers. The fluorescence anisotropy readout reveals the system's reactivity to small‐molecule targets, offering the unique ability to provide both enhanced and consistent responses across a range of high serum levels (25%–90%). As the sensor's performance may be sensitive to inter‐sample serum variations, a standard addition calibration approach is used to ensure reliable quantitative analyses. Overall, the proposed strategy addresses substantial serum‐induced perturbations and preserves assay functionality under biologically challenging conditions, thereby establishing a practical framework for extending homogeneous aptamer‐based sensing to clinically relevant blood matrices.

## Graphical abstract

The sample self‐analyzes: The predominant serum proteins, albumin and immunoglobulin G, were leveraged to activate the response of fluorescent aptamer probes within the biological matrix. The fluorescence‐anisotropy‐based sensing platform demonstrated both performance and consistency for the direct detection of small molecules in serum‐rich samples, highlighting its potential to advance homogeneous‐phase aptamer assays in blood fluids.

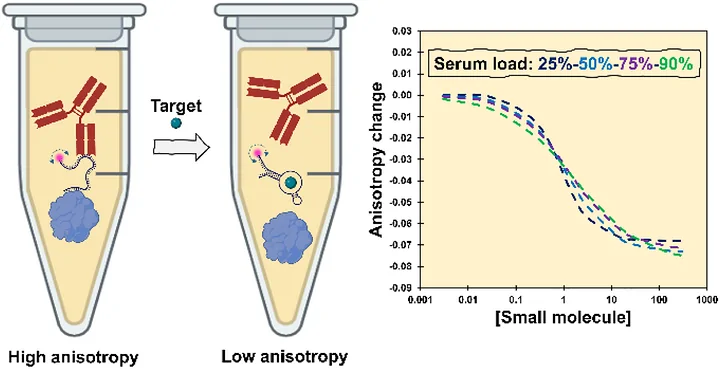

## 1 Introduction

In the fast‐growing area of aptamer‐based technology [1, 2], a huge variety of sensors have been designed for quantifying low‐molecular‐weight analytes of importance to the environmental [3], agri‐food [4], and health [5] sectors. In the latter, the measurement of metabolites, drugs, illicit drugs, or contaminants in human serum, an essential matrix in clinical chemistry and toxicology [6], is of paramount value for biomedical diagnosis, therapeutic drug monitoring, assessment of exposure to hazardous products, forensic medicine, and anti‐doping control.

Thanks to their simple “mix‐and‐measure” operation and their suitability for high‐throughput analysis, homogeneous aptamer sensors, in particular those based on fluorescence readout, are attracting considerable attention for the detection of small‐molecule targets [7, 8, 9, 10, 11, 12]. However, their direct transposition from the buffer to serum specimens remains a real challenge. Autofluorescence and light scattering [13, 14], along with non‐specific binding of endogenous molecules to sensor components [15, 16, 17], cause poorly controllable disruptions as biofluid content rises, potentially leading to total signal loss or even reversal (see Table S1 for data collected from more than 50 studies).

This study examines a sensing approach developed to operate preferentially in serum environments, employing simple fluorophore‐labeled aptamers as probes (Figure 1). The method no longer views the biological matrix as a source of interference for a pre‐designed sensor, but rather as an integrated driving component of the system. To some extent, this echoes a concept previously described in intracellular fluorescence imaging, where small‐dye‐binding properties of cytoplasmic proteins, typically considered undesirable, are instead leveraged to improve the signal in situ [18, 19]. Serum proteins are well known to adsorb non‐specifically onto DNA molecules [20, 21, 22, 23], a phenomenon regularly associated with matrix effects in aptamer assays [16, 17, 24]. Interestingly, certain serum proteins also appear to distinguish between ssDNA and dsDNA [23], a feature that may aid in discriminating between the unfolded and folded states of the aptamer [25, 26]. Herein, we showed that endogenous albumin and immunoglobulin G can trigger the aptamer probe response within the biofluid (Figure 1). This approach proved suitable for directly sensing small molecules in serum‐rich samples, as demonstrated across seven targets, while addressing assay‐impairing perturbations.

**FIGURE 1:**
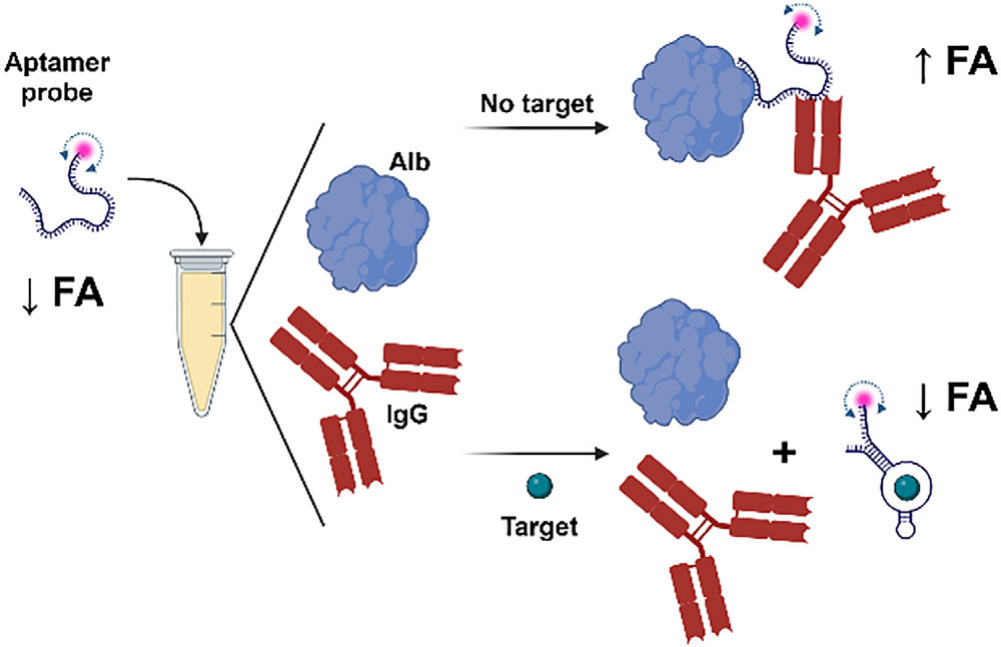
In routine practice, homogenous sensors are initially developed in a bare buffer. When faced directly with a serum sample, these regularly show substantial perturbations in performances, with the biofluid fraction increasing. Here, the biological matrix inherently serves as the driving component of the sensor, offering advantageous detection in serum‐rich specimens. The predominant endogenous proteins, such as albumin (Alb) and immunoglobulin G (IgG), are capable of triggering the sensing event. The introduction of an aptamer probe into a target‐free serum sample leads to the in situ formation of complexes with proteins, resulting in a mass‐dependent increase in fluorescence anisotropy (FA). In the presence of the small analyte, these assemblies are competitively prevented by the target binding to the aptamer, which leads to an increased fraction of the non‐protein‐bound probe and thus a target‐dependent reduction in FA.

## 2 Results and discussion

To track changes in the binding state of aptamer probes during their association with matrix proteins, while monitoring potential alteration induced by the small target, we naturally turned to fluorescence anisotropy (FA) as a readout technique. This constitutes the gold standard in fluorescence for capturing in solution any fluctuation in the apparent molecular mass of a small fluorescent ligand as it interacts with macromolecules [27], also providing reliable sensing platforms [10, 16, 26, 27]. We first focused on the L‐tyrosinamide‐binding DNA aptamer [28] (apt‐Tym) used to develop proof‐of‐principle sensors and enzyme mimicry systems [9, 10, 26, 29]. In order to reduce serum autofluorescence interferences, the red‐shifted fluorophore Texas Red (TR, *λ*_exc_: 590 nm and *λ*_em_: 635 nm) was covalently attached to the 3’ end of the apt‐Tym sequence to serve as the probe. Because of its availability in large volumes, commercial samples of pooled human serum were initially favored to assess the matrix's activating potential toward the aptamer probe.

We first titrated 3'‐TR‐apt‐Tym with increasing fractions (F) of serum, with a maximum of 90% matrix, to allow reliable introduction of the assay components into the working solution. We recorded a regular increase in the FA signal, reaching up to 0.140 in 90% biofluid (Figure 2a). This is consistent with previous results for other functional oligonucleotides, suggesting an association with serum proteins [16]. Addition of L‐tyrosinamide (L‐Tym) at the 300 µM saturating concentration led to a downward shift in the probe‐matrix binding curve compared with that recorded in the absence of target (Figure 2a). Control experiments using either non‐cognate enantiomer (D‐Tym) or mutated aptamer (3’‐TR‐apt_mut_‐Tym) proved that the offset of the binding curve toward higher serum fractions depends solely on the binding of the DNA receptor to its target (Figure 2a), acting as a competitive event with respect to its interaction with endogenous biomolecules.

**FIGURE 2:**
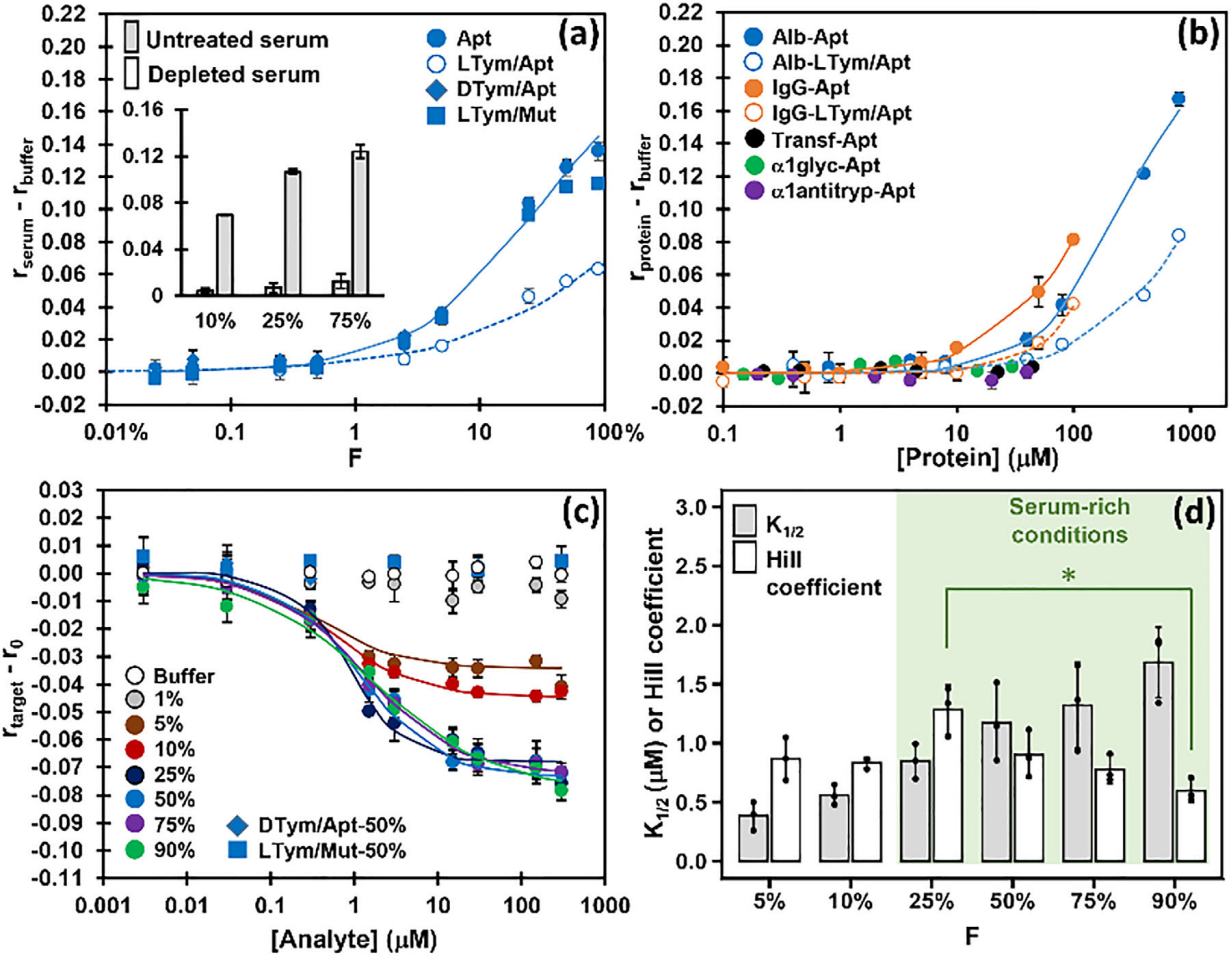
(a) FA change of 3’‐TR‐apt‐Tym versus F of untreated pooled serum, without or with 300 µM L‐Tym. Control data using 3’‐TR‐apt_mut_‐Tym in the presence of 300 µM L‐Tym or 3’‐TR‐apt‐Tym in the presence of 300 µM D‐Tym are provided. r_buffer_ is the FA in clean buffer, whereas r_serum_ is the FA with added biofluid. Inset: Comparison of 3'‐TR‐apt‐Tym binding to albumin/IgG‐depleted serum versus binding to untreated serum. (b) FA change of 3’‐TR‐apt‐Tym versus protein concentration (albumin, IgG, transferrin, α1‐acid glycoprotein and α1‐antitrypsin), in the absence of L‐Tym (and with 300 µM target for albumin and IgG). r_protein_ is FA with increasing protein. (c) L‐Tym‐dependent FA response curve using 3’‐TR‐apt‐Tym in clean buffer and pooled serum (*F* = 1%–90%). Same control data as in (a) for F = 50%. r_target_ is FA with increasing target and r_0_ is FA without target. (d) K_1/2_ and Hill coefficient determined by fitting the Hill model to experimental data from (c). At 5%–10% serum, the *K*_1/2_ values were similar to the dissociation constant of the native complex [28]. *K*_1/2_ and Hill coefficient values were compared across the relevant 25%–90% serum content range using Welch's ANOVA with Games‐Howell post hoc testing (*p < 0.05). Error bars represent SD from three independent experiments.

To gain insight into the origin of this responsivity, we performed similar DNA‐binding experiments to some of the major proteins present in the biofluid, taken individually. Human serum albumin, immunoglobulin G (IgG), transferrin, α1‐antitrypsin, and α1‐acid glycoprotein were employed under buffered conditions. For each of them, the concentration range tested was chosen to reach the upper limit of its physiological level [30]. It was found that only albumin and, to a lesser extent, IgG bind to free aptamer in their concentration window (Figure 2b). Following the trend observed in serum, the formation of these aptamer‐protein complexes was prevented upon addition of 300 µM L‐Tym (Figure 2b), demonstrating the ability of albumin and IgG to turn on the sensor on their own. To further assess their role, we performed a binding assay on pooled serum, but from which the two proteins were specifically removed by a pretreatment with a mixed bed of Cibacron blue/protein A gel [31]. A slight rise in FA was observed as the proportion of depleted serum increased (Figure 2a), representing only about 10% of the variation seen in the untreated matrix at *F* = 75%. This suggests that probe binding, and thus its activation, is primarily driven by the two predominant blood proteins. Although some other serum proteins may also bind significantly to nucleic acids [32], their relatively low abundance in the biofluid likely reduces their overall impact.

We then set out to construct target‐dependent FA response curves in the different portions of pooled serum. Preliminary experiments carried out in clean buffer did not produce any response for 3’‐TR‐apt‐Tym (Figure 2c). Increasing biofluid content resulted in a target‐dependent decrease in FA, which became detectable at matrix levels above 1%. The largest signal variation was observed under serum‐rich conditions (*F* = 25%–90%), where values reached 0.070–0.080 (Figure 2c and Table S2). In this range, K_1/2_ values showed only a moderate increase relative to those measured at 5%–10% matrix (Figure 2d). Accordingly, operation under elevated biological loads provides a favorable balance between signal gain and apparent affinity, while yielding broadly comparable sensor response profiles (Figure 2c,d). Moreover, the limit of detection achieved at the highest matrix level, namely 73 nM determined from 3σ of the blank, is lower than many of the values obtained with other homogeneous fluorescent sensors operated in a clean buffer [9, 10, 26]. Control experiments confirmed that the sensor is both specific and selective (Figure 2c). Replacing the TR fluorophore with an even more red‐shifted label (Alexa Fluor 647, *λ*_exc_: 647 nm and *λ*_em_: 692 nm) also resulted in an output signal, but with much lower assay performance (Figure S1).

Once the feasibility study with the L‐Tym aptamer was completed, we considered extending the strategy to other aptamers. We focused on classic DNA receptors specific for cocaine (Coc) [33] and adenosine (Ade) [34], along with more recent aptamers derived from the widely used capture‐SELEX method, targeting theophylline (Theo) [35], L‐tryptophan (L‐Trp) [36], dopamine (Dpm) [5], and methotrexate (Mtx) [37]. Previously reported truncated variants, which promote unfolding in the free state and enhance target‐induced structural changes [36, 38, 39, 40], were favored. The TR conjugates remained largely inactive in the clean buffer. Conversely, when evaluated in serum‐rich samples (Figures S2 and S3 and Table S2), these probes showed a specific and selective response (Figure 3). The sensors exhibited K_1/2_ values that are fairly close to the dissociation constants of the target/variant complexes [36, 38, 39, 40].

**FIGURE 3:**
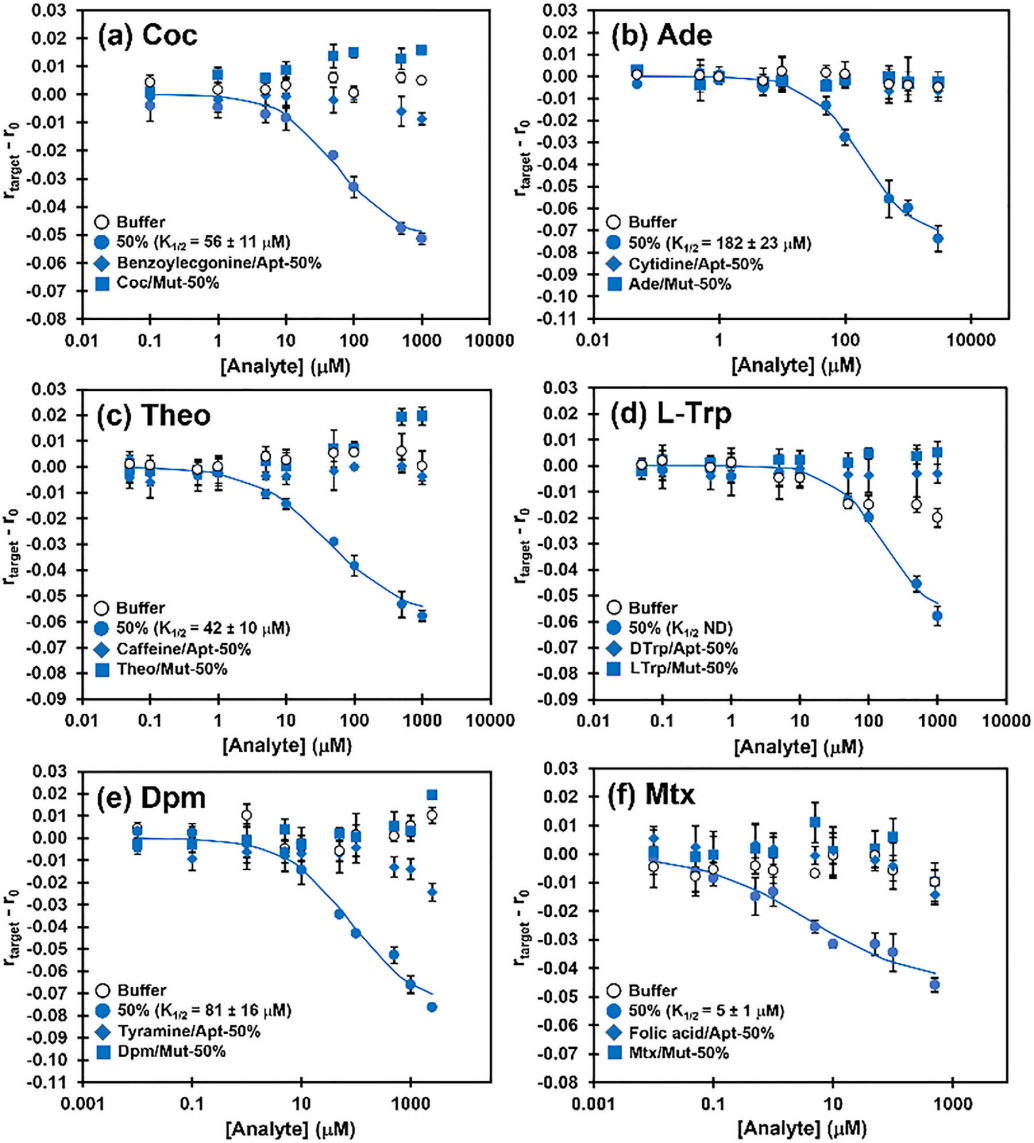
Target‐dependent FA response curve in clean buffer and 50% pooled serum for (a) Coc using 5’‐TR‐apt‐Coc, (b) Ade using 3’‐TR‐apt‐Ade, (c) Theo using 5’‐TR‐apt‐Theo, (d) L‐Trp using 5’‐TR‐apt‐Trp (K_1/2_ was not determined, as L‐Trp is 20–100 µM in blood [36]), (e) Dpm using 5’‐TR‐apt‐Dpm and (f) Mtx using 5’‐TR‐apt‐Mtx. Control measurements obtained with the corresponding mutated aptamers and non‐cognate ligands are shown for *F* = 50%. r_target_ is FA with increasing target, and r_0_ is FA without target. The error bars represent SD from 3 independent experiments.

We then sought to evaluate the relevance of this approach by comparing it with a long‐established aptamer sensing strategy under serum exposure. We constructed an L‐Tym sensor based on the design of structure‐switching aptamers [2], also referred to as duplexed aptamers [41]. First, we used the same FA readout protocol, allowing an analogous target‐induced decrease in response [42, 43] (Figure 4a). The labeling strategy involved conjugating the TR label to a 10‐mer complementary strand (5'‐TR‐CS‐Tym) to facilitate the mass‐based output signal. In clean buffer, the formation of the CS‐aptamer duplex led to an increase in FA due to a rise in the effective dimension of 5'‐TR‐CS‐Tym. The binding of L‐Tym to apt‐Tym competed with hybrid formation, thereby increasing the amount of free probe and causing the signal reduction. The increase in biofluid fractions resulted in continuously higher FA values in the absence of target, reflecting the binding of matrix proteins to the duplex (Figure S4). This was accompanied by a near‐total loss of sensor response at just 5% serum (Figure 4a). We next implemented the most commonly used signaling mode, based on fluorescence intensity (FI) [8, 11, 12, 13, 17, 37, 41]. To retain the same DNA strand as a probe, CS‐Tym was conjugated at its 3’ end with the TR fluorophore, while apt‐Tym served as the quencher via 5’‐BHQ2 labeling [44]. Consistent with the above principle, the FI signal increased upon target addition to the clean buffer (Figure 4b). Although the FRET‐based sensor demonstrated improved resistance to matrix interference, a significant increase in background noise (Figure S4) compromised its response, making detection challenging or even impractical in 75%–90% serum (Figure 4b). Irrespective of the mode used, the results corroborate general observations that duplexed aptamer sensors gradually deteriorate as serum content increases (Table S1), in stark contrast to the present test (Figure 2c).

**FIGURE 4:**
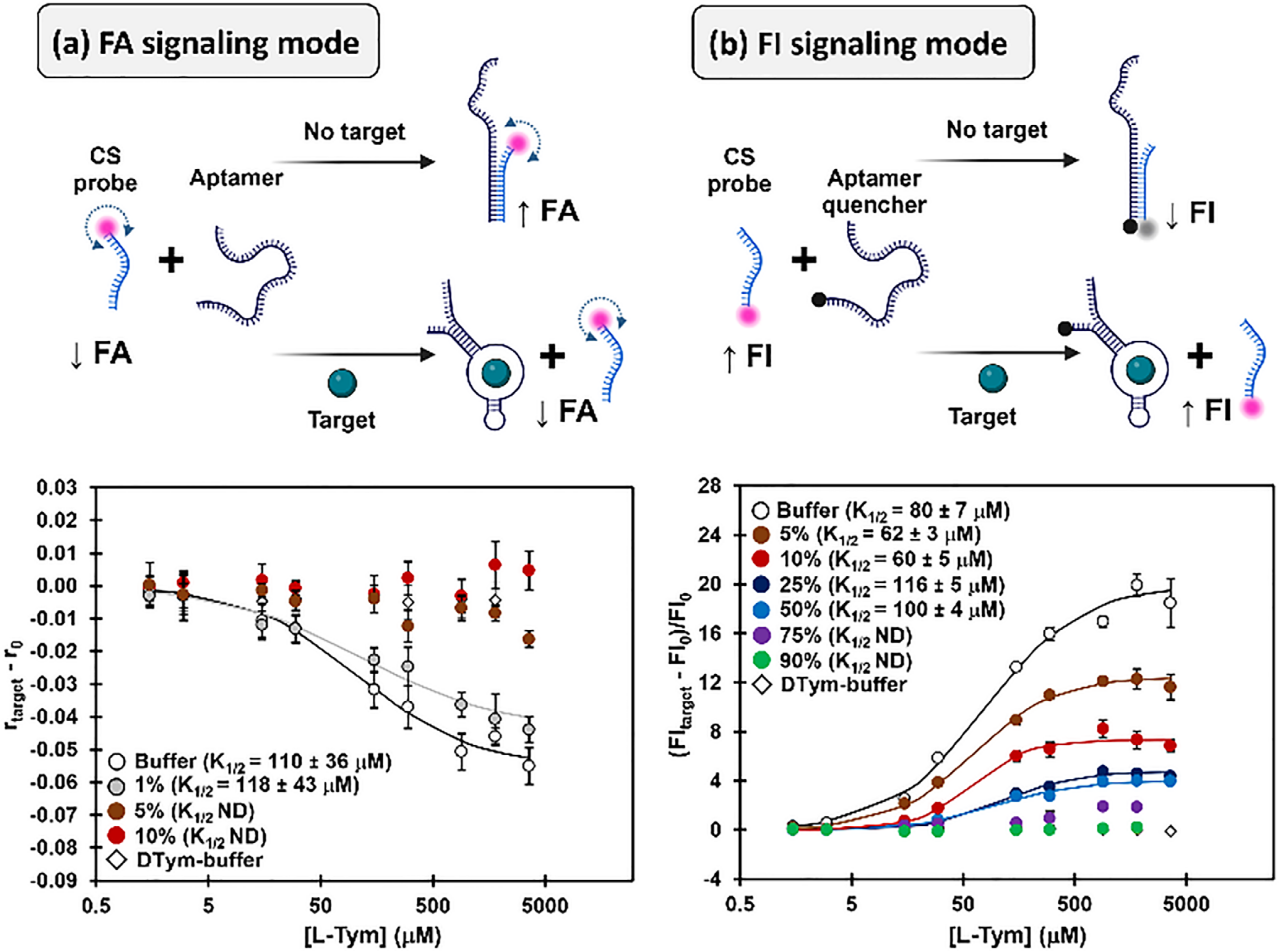
(a) Schematic of the FA‐based duplexed aptamer sensor with the associated L‐Tym‐dependent response curve (1:1 stoichiometry for the 5'‐TR‐CS‐Tym:apt‐Tym hybrid) in clean buffer and pooled serum (*F* = 1%–10%). r_target_ is FA with increasing target and r_0_ is FA without target. (b) Schematic of the FI‐based duplexed aptamer sensor [44] with the associated L‐Tym‐dependent response curve (1:1 stoichiometry for the 3’‐TR‐CS‐Tym:5'‐BHQ2‐apt‐Tym hybrid) in clean buffer and pooled serum (*F* = 5%–90%). FI_target_ is the signal with increasing target and FI_0_ is the signal without target. Control experiments using D‐Tym under buffer conditions are also depicted in both cases. The error bars represent SD from 3 independent experiments.

We subsequently assessed whether the sensing scheme could satisfy the requirements for practical operation. First, the implementation of the matrix‐activated system is expected to be compromised if substantial dilution of the sample is required, that is, when analyte concentrations exceed the dynamic range of the sensor. One obvious solution would be to lower the final target concentrations by diluting the specimen with a comparable proportion of blank serum matrix, i.e., free of the target. Since this approach is, in principle, only feasible for analytes that are not endogenous to blood, we instead proposed a closely related yet generalizable alternative, which consisted of supplementing the further diluted sample with external albumin in order to recover sensor response curves aligned with relevant concentrations (Figure S5).

In addition, the pooled serum we analyzed may not fully capture the complexity and variability seen at the individual level, regarding both naturally occurring blood components and exogenous substances. The sensor was thus tested on serum specimens from a diverse cohort of subjects. These samples were selected with a particular focus on the matrix activating elements, thereby covering a wide range of concentrations of total proteins, albumin, and γ‐globulins (Table S3 and Figure 5a). Despite some variations in the target‐dependent response curve, inherent to the matrix‐related nature of the sensing principle, the L‐Tym sensor consistently demonstrated the expected behavior across all specimens (Figure 5a). To further investigate, we studied the influence of a representative set of drugs on the output signal of 3'‐TR‐apt‐Tym in pooled serum. More specifically, we focused on compounds known to strongly bind serum proteins to assess their potential to interfere with the probe for matrix interactions (Figure S6). It was shown that the FA value remains unaffected within the therapeutic blood concentrations of the tested drugs (Figure S6).

**FIGURE 5:**
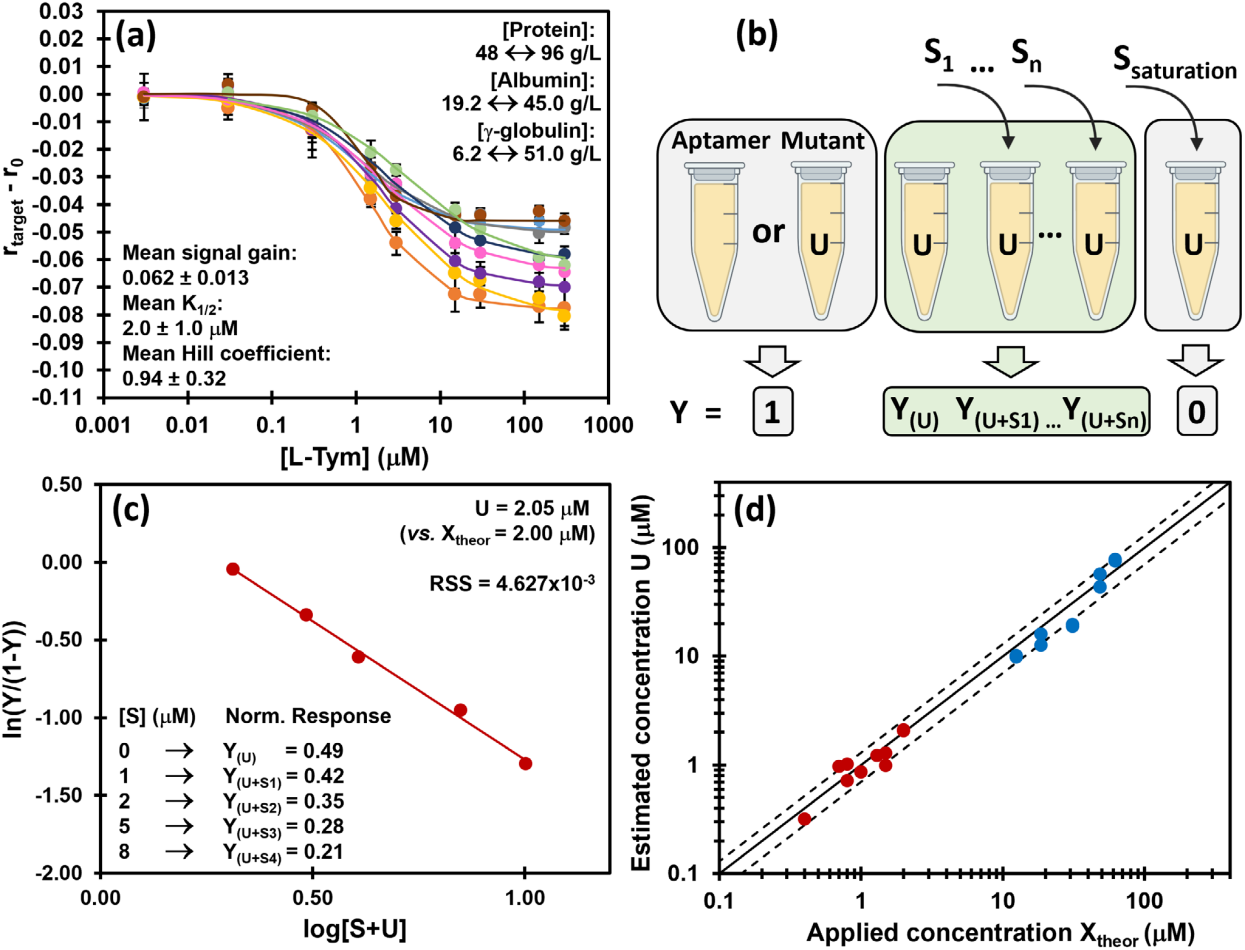
(a) L‐Tym‐dependent FA response curves using 3'‐TR‐apt‐Tym tested on 50% serum samples from nine different subjects. *r*_target_ is FA with increasing target and *r*_0_ is FA without target. The error bars represent SD from 3 independent experiments. (b) Schematic of the SAM calibration. S is the concentration of the target analyte added to aliquots of the serum sample containing an unknown concentration (*U*). *Y* is the normalized response, ranging from 1 in the absence of target, which could also be assessed using a mutated aptamer in the presence of target, to 0 when the analyte is at saturation. (c) Calculation of U using the SAM calibration with data linearization for L‐Tym sensor (50% pooled serum). In the Excel spreadsheet provided in Supporting Information, the mean *Y* value from 3 independent measurements [45, 46] was first subjected to a logit transformation according to the relation ln(*Y*/(1–*Y*)). These values were reported as a function of the logarithm of the sum of the addition (S_1_–S_4_) and *U*. Using the Solver tool, *U* was determined by minimizing the linearity deviation, as indicated by the lowest residual sum of squares (RSS), yielding the calibration line shown. (d) Estimated concentration *U* plotted as a function of the applied concentration *X*_theor_ using the L‐Tym (pooled sera, *n* = 10, red circle) and Theo (individual sera, *n* = 10, blue circle) sensors. The solid line represents the *Y* = *X* correlation, and the dashed lines the ±30% accuracy margin [47].

To best manage the inter‐sample variability in quantitative analysis, we naturally resorted to the standard addition method (SAM), which mitigates the influence of complex matrices by performing calibration directly within each specimen [45, 46, 47] (Figure 5b). We specifically applied the logit‐log linearization tailored for bioassays with sigmoidal responses [47]. Using this SAM protocol (Figure 5c), sensor performance was evaluated with both pooled and individual serum samples spiked with known target concentrations (*X*_theor_) (Figure 5d). The overall accuracy and precision (Table S4) were comparable to or better than those reported for several heterogeneous‐phase systems tested under similar conditions [48, 49, 50, 51, 52, 53, 54] (Table S5).

## 3 Conclusion

In summary, we have developed a homogeneous aptamer assay strategy designed for the detection of small molecules under high‐serum‐load conditions. By its very nature, this approach cannot overcome all sources of performance variability associated with this biological environment. In particular, *K*_1/2_, the Hill coefficient, and the FA signal gain may be sensitive to sample‐to‐sample variations in serum content and composition. SAM‐based calibration enables these effects to be accounted for, thereby supporting reliable quantitative analyses.

By addressing major serum‐induced disturbances, including substantial losses in affinity and response or even complete signal suppression, the proposed method provides a practical framework for solution‐phase analysis under biologically challenging conditions. Its successful use with various aptamers, including those derived from the increasingly popular capture‐SELEX approach, further underscores its potential for broader application.

## Author Contributions

**Amandine Barbaroux**: writing – original draft, investigation, data curation, methodology, validation, software. **Benoit Chovelon**: conceptualization, funding acquisition, writing – review and editing, investigation, supervision. **Emmanuelle Fiore**: investigation, methodology. **Sebastien Vezirian**: investigation, methodology. **Mickael Henry**: investigation, methodology. **Marwa Zoubeidi**: investigation, methodology. **Maria C. Derosa**: funding acquisition, writing – review and editing, supervision. **Corinne Ravelet**: supervision, conceptualization, funding acquisition, writing – review and editing. **Eric Peyrin**: project administration, conceptualization, funding acquisition, writing – original draft, writing – review and editing, supervision, validation, software.

## Conflicts of Interest

The authors declare no conflicts of interest.

## Supporting information




**Supporting File**: The authors have cited additional references within the Supporting Information [15, 26, 27, 28, 36, 37, 38, 39, 40, 45, 47, 48, 49, 50, 51, 52, 53, 54, 55, 56, 57, 58, 59, 60, 61, 62, 63, 64, 65, 66, 67, 68, 69].

**Supporting File**: anie74517‐sup‐0002‐SuppMat.pdf.

## Data Availability

The data that support the findings of this study are available from the corresponding author upon reasonable request.
