# Supplementary material for: Matrix‐Activated Aptamer Sensors for Human Serum Analysis
Source: Angew Chem Int Ed Engl. 2026 Sep 2;65(42):e3409471. doi: 10.1002/anie.3409471 (PMC13653630; doi:10.1002/anie.3409471)
Supplement: Supplementary file 1 [file ANIE-65-e3409471-s002.pdf]

Table of Contents

Experimental section (S2)
- I. Reagents (S2)
- II. Human serum sourcing from individuals (S2)
- III. Sequences of DNA oligonucleotides (S2)
- IV. Measurements and data analysis (S2)
- V. Experimental procedures (S3)

Tables S1-S5 (S6-S11)
Figures S1-S7 . (S12-S18)

References (S19)
Author contributions (S19)

Experimental section

I. Reagents

Pooled human serum (batches: 0000303263, 0000399415, 0000298223, 0000326799 and 304064), Human serum albumin HSA (batches: 120K7603 and 0000415243), transferrin (batch: 0000314811), α1-antitrypsin (batch: 0000479204), immunoglobulin G IgG (batch: BCCK3933), α1-acid glycoprotein (batch: 0000332583), Tris base, L-tyrosinamide (L-Tym), caffeine, adenosine, cytidine, dopamine (Dpm), tyramine, methotrexate (Mtx), folic acid, acetylsalicylic acid, phenytoin, warfarin, were purchased from Sigma-Aldrich (Saint-Quentin-Fallavier, France). L-tryptophan (L-Trp) and D-tryptophan (D-Trp) were provided by Alfa Aesar (Kandel, Germany). D-tyrosinamide (D-Tym) was ordered from Iris Biotech (Marktredwitz, Germany). Theophylline (Theo) was purchased from TCI Europe N.V. (Zwijndrecht, Belgium). Cocaine (Coc) and benzoylecgonine were supplied by Lipomed AG (Arlesheim, Switzerland). Quinine was purchased from Cooper (Nozay, France). Ibuprofen was provided by Fagron (Nazareth, Belgium). Magnesium chloride ($MgCl_2$), sodium chloride (NaCl), diazepam and Pierce Albumin/IgG removal kit were obtained from Thermo Fisher Scientific (Geel, Belgium or Rockford, USA). Water was obtained using a Direct-Q® 3UV water purification system (Merck Millipore, Guyancourt, France). Serum samples were aliquoted and stored at -20°C until use. Stock solutions of proteins and most analytes were prepared in pure water. Exceptions included MTX, which was prepared in 0.1 M NaOH, and phenytoin, quinine, ibuprofen, and diazepam, whose stock solutions were prepared in the presence of DMSO. The binding buffer was prepared to desired pH (7.5) by titrating the free base with hydrochloric acid. DNA oligonucleotides were synthesized, HPLC-purified and identified by mass spectrometry by Eurogentec (Seraing, Belgium). The stock solutions of oligonucleotides were prepared in sterile water Carl Roth (Karlsruhe, Germany) at 10^{-3} M and stored at -20°C until use.

II. Human serum sourcing from individuals

The non-interventional, monocentric, retrospective study involving data and serum samples from human participants was carried out at Grenoble University Hospital according to French current regulation. The investigators signed a commitment to comply with Reference Methodology n°004 issued by French Authorities (CNIL). Subjects were all informed and did not oppose; written consent for participation was not required for this study in accordance with the national legislation and the institutional requirements. The raw data will be made available by the authors within respect of General Data Protection Regulation, without undue reservation.

III. Sequences of DNA oligonucleotides

The truncated aptamers used in this study correspond to the following sequences: 23-mer oligonucleotide binding to L-Tym,[S1] 21-mer oligonucleotide binding to Ade,[S2] MN19,[S3] Th2201-t2,[S3] TRNC17/11,[S4] DA-mut3-del6,[S5] and HMX24.[S3] These are hereinafter referred to as apt-Tym, apt-Ade, apt-Coc, apt-Theo, apt-Trp, apt-Dpm, and apt-Mtx, respectively. To perform control experiments with a non-binding oligonucleotide, we used a mutated sequence rather than a scrambled sequence in order to maintain the same overall DNA structure. To select these sequences, we proceeded depending on the aptamer sensor. We introduced random point mutations at two positions (G2C and G4C) at the 5′ end of apt-Tym, given that the integrity of the full 23-mer sequence is critical for target binding.[S1] The G6C and G19C mutations were incorporated into the apt-Ade sequence due to the involvement of both G bases in the target's binding sites.[S6] Regarding the Coc-binding aptamer, we introduced T16C and G27C mutations in the sequence on the basis of the NMR analysis of the complex.[S3] For apt-Theo, we employed the non-binding T19C mutant derived from crystallographic studies of the Theo–aptamer complex.[S7] In the case of apt-Trp, two nucleotides were randomly mutated (G18C and G20C) in the apical loop, as this motif plays a key role in L-Trp recognition.[S4] Guided by NMR analysis of the binding pocket,[S8] we introduced C20T and G27T mutations into the apt-Dpm sequence. The G5T mutation was incorporated into the apt-Mtx sequence as described by Shi's laboratory.[S9]

Oligonucleotide name	Length	Sequence (5' → 3')
apt-Tym	23-mer	TGTGGTGTGTGAGTGCGGTGCCC
3'-TR-apt-Tym	23-mer	TGTGGTGTGTGAGTGCGGTGCCC-TR
5'-BHQ2-apt-Tym	23-mer	BHQ2-TGTGGTGTGTGAGTGCGGTGCCC
5'-AF647-apt-Tym	23-mer	AF647-TGTGGTGTGTGAGTGCGGTGCCC
3'-TR-apt$_{mut}$-Tym	23-mer	T**C**T**C**GTGTGTGAGTGCGGTGCCC-TR
5'-TR-CS-Tym	10-mer	TR-ACACACCACA
3'-TR-CS-Tym	10-mer	ACACACCACA-TR
5'-TR-apt-Coc	30-mer	TR-GACAAGGAAAATCCTTCAACGAAGTGGGTC
5'-TR-apt$_{mut}$-Coc	30-mer	TR-GACAAGGAAAATCCT**C**CAACGAAGTG**C**GTC
3'-TR-apt-Ade	21-mer	TGGGGGAGTATTGCGGAGGAA-TR
3'-TR-apt$_{mut}$-Ade	21-mer	TGGGG**C**AGTATTGCGGAG**C**AA-TR
5'-TR-apt-Theo	23-mer	TR-GTGGTCTATTCATAGGCGTCCGC
5'-TR-apt$_{mut}$-Theo	23-mer	TR-GTGGTCTATTCATAGGCG**C**CCGC
5'-TR-apt-Trp	29-mer	TR-CCGGTGGTGTAGTTCCGGCGTGGGGAAGG
5'-TR-apt$_{mut}$-Trp	29-mer	TR-CCGGTGGTGTAGTTCCG**C**C**C**TGGGGAAGG
5'-TR-apt-Dpm	32-mer	TR-CAGTTTGAAGGTTCGTTCGCAGGTGTGGAGTG
5'-TR-apt$_{mut}$-Dpm	32-mer	TR-CAGTTTGAAGGTTCGTTCG**T**AGGTGT**T**GAGTG
5'-TR-apt-Mtx	24-mer	TR-CGCGGGATGTTTGGGGGACCCACG
5'-TR-apt$_{mut}$-Mtx	24-mer	TR-CGCG**T**GATGTTTGGGGGACCCACG

The end-attached fluorophores or quenchers are shown in red (TR: Texas Red; AF647: Alexa Fluor 647; BHQ2: Black Hole Quencher 2). The mutations in control oligonucleotides are bold and green colored.

IV. Measurements and data analysis

Total protein concentrations were measured using the Siemens Atellica® Solution analyzer (Siemens Healthcare Diagnostics, New York, USA). Albumin and γ-globulin concentrations were most often determined using the capillary electrophoresis system with UV detection Capillarys 3 Octa® (Sebia, Lisses, France), with the exception of certain albumin assays, which were also performed on the Atellica® Solution analyzer.

Fluorescence anisotropy (FA) and fluorescence intensity (FI) readings were taken on a Tecan Spark microplate multimode reader (Männedorf, Switzerland) using black, 96-well Greiner Bio-One microplates (Courtaboeuf, France). Excitation was set at 590 nm for Texas Red conjugates and 647 nm for the Alexa Fluor 647 conjugate, and emission was recorded at 635 nm and 692 nm, respectively. The FA value (r) was determined by the instrument software SparkControl. The reported mean values and standard deviations were from independent experiments performed in triplicate.

When analyzing probe interaction with serum matrix (or individual proteins), FA change referred to r_{serum} (or $r_{protein}$) - r_{buffer} where r_{buffer} is the FA signal in clean buffer and r_{serum} (or $r_{protein}$) is the FA signal in the presence of increasing biofluid fraction (or protein concentration). Since no binding saturation was reached under the experimental conditions for the binding curves of 3'-TR-apt-Tym with albumin and IgG (Figure 2b), we were unable to accurately determine their midpoint.

For target-dependent FA response data obtained using either matrix-activated or duplexed aptamer sensors, FA change (Δr) corresponded to r_{target} - r_0, where r_{target} is the FA signal in the presence of increasing target concentration and r_0 is the FA signal in the absence of target. The fitting procedure was completed with a R program using nonlinear least squares (nls) function by analyzing each curve from each replicate. The following Hill model was used to fit the experimental data of the target-binding curves:

$$\Delta r = \Delta r_{max} \times \frac{c^n}{K_{1/2}^n + c^n}$$

where Δr_{max} is the Δr value at maximum target concentration (signal gain), $K_{1/2}$ the target concentration needed to obtain 50% of the signal gain, c the target concentration and n the Hill coefficient. Statistical comparisons of $K_{1/2}$ and Hill coefficient values in the relevant serum range of 25% to 90% were performed using Welch's ANOVA followed by Games-Howell post hoc testing. Statistical significance was defined as $p < 0.05$.

For target-dependent FRET response data obtained using a duplexed aptamer sensor, FI change (ΔFI) corresponded to FI_{target} - FI_0, where FI_{target} is the FI signal in the presence of increasing target concentration and FI_0 is the FI signal in the absence of target. We used the same Hill model for curve fitting:

$$\frac{\Delta FI}{FI_0} = \frac{\Delta FI_{max}}{FI_0} \times \frac{c^n}{K_{1/2}^n + c^n}$$

where ΔFI_{max} is the ΔFI value at maximum target concentration.

The Z-prime (Z') value was determined as follows:[S10]

$$Z' = 1 - \frac{(3\sigma_0 + 3\sigma_{target-max})}{\left|\mu_0 - \mu_{target-max}\right|}$$

where μ_0 and σ_0 represent the mean FA signal and its standard deviation in the absence of target, respectively, and $\mu_{target-max.}$ and $\sigma_{target-max}$ are the mean FA signal and its standard deviation at maximum target concentration, respectively.

The calculation of target concentration U through the standard addition method (SAM) was carried out using an Excel spreadsheet from a previous work,[S11] which was modified to include the improved logit-log linearization described by the Gauglitz group.[S12] This is provided as a separate Supporting Information file ("SI_matrix_SAM"). In line with standard practice in calibration procedures, this SAM approach also relied on linear regression based on the average response obtained from 2 to 3 independent measurements.[S11]

V. Experimental procedures

General considerations

For simplicity, the binding buffer selected for the L-Tym system[S1] was initially utilized for the other sensors (Figure S2). The $MgCl_2$ concentration was subsequently adjusted on a case-by-case basis to improve the signal gain, as illustrated in Figure S3. The buffer conditions presented below are those that were ultimately chosen. The serum experiments were conducted while maintaining the same final concentration of binding buffer in the wells, regardless of the matrix fraction or sample type. Accordingly, the assay components intended for the final working solution were prepared in a buffer solution whose concentration was adjusted according to the serum fraction used (for example, for samples containing 50% serum in the final solution, they were first reconstituted in 2X binding buffer before being added to the wells).

The probe solutions were heated to 80°C for 5 minutes, then left at room temperature (RT) in the dark (final concentration of 80 nM, except for the AF647 probe, which was 160 nM). A volume of 100 µL was added to each well, either buffer for the control or a mixture containing the assay components (e.g., oligonucleotides, targets, and serum or individual proteins). For each type of experiment

conducted, a preliminary analysis was carried out to verify that the measurement on the microplate reader remained stable over time, indicating that equilibrium was already reached after mixing the different solutions in the wells.
The conditions of use for the systems studied are summarized below:

System	Binding buffer	Non-cognate ligand	Batch of pooled human serum
L-Tym*	10 mM Tris pH 7.5, 50 mM NaCl, 10 mM $MgCl_2$	D-Tym	0000303263, 0000399415, 000298223, 0000326799, 304064
Coc	10 mM Tris pH 7.5, 50 mM NaCl	Benzoylecgonine	0000298223
Ade	10 mM Tris pH 7.5, 50 mM NaCl, 0.5 mM $MgCl_2$	Cytidine	0000326799
Theo**	10 mM Tris pH 7.5, 50 mM NaCl, 0.5 mM $MgCl_2$	Caffeine	0000303263
L-Trp	10 mM Tris pH 7.5, 50 mM NaCl, 0.5 mM $MgCl_2$	D-Trp	0000399415
Dpm	10 mM Tris pH 7.5, 50 mM NaCl, 10 mM $MgCl_2$	Tyramine	0000298223
Mtx	10 mM Tris pH 7.5, 50 mM NaCl, 2 mM $MgCl_2$	Folic acid	0000298223

*For the analysis of individual samples in Figure 5a, the NaCl concentration was increased to 75 mM to better reflect physiological Na^+ levels at F = 50%. Since this adjustment had only a minimal effect on the overall sensor response, it was not retained for subsequent SAM-based quantitative analyses (Figure 5d and Table S4).
**The sensor's dependence on the concentration of divalent cations (Figure S3) was utilized to match its response to clinically relevant concentrations of Theo in SAM-based quantitative analyses (Figure 5d and Table S4), by setting the operating point at 0.5 mM Mg^{2+}.

Probe interaction with human serum
Two aptamer working solutions were prepared as above mentioned, without or with L-Tym (final concentration: 300 µM). Successive dilutions of serum were made, from 0 to 90% final fraction. Aptamer and serum working solutions were then mixed into individuals wells. Control experiments using mutated aptamer probes and non-cognate ligands were performed following the same protocol. Similar experiments were achieved for other aptamer systems, except than serum range was from 0 to 50% at final fraction.
For experiments conducted on serum samples (50% final fraction) enriched with protein-binding drugs (Figure S6), the same protocol was applied for the 3'-TR-apt-Tym probe, with the exception that each drug was introduced at varying concentrations into each well.

Probe interaction with Individual serum proteins
The same experimental procedure was used for all proteins, namely HSA, IgG, transferrin, α1-acid glycoprotein and α1-antitrypsin. As described above, two aptamer working solutions were made, with or without L-Tym (final concentration: 300 µM). Successive protein dilutions were prepared as follows: 0-800 µM for HSA, 0-100 µM for IgG, 0-45 µM for transferrin, 0-30 µM for α1-acid glycoprotein and 0-40 µM for α1-antitrypsin. Aptamer and protein working solutions were then mixed into individuals wells.

Probe interaction with Albumin/IgG-depleted human serum
The protocol for depletion was adapted from supplier instruction. Especially, the buffer used was the binding buffer of L-Tym system. First step was resin preparation: 170 µL of mixed bed of Cibacron blue/protein A gel was added on spin columns. Spin columns were then placed in microcentrifuge collection tubes and centrifuge at 10,140 rpm for 1 min. Storage buffer was discarded and plugs was inserted into bottom of each columns containing the gel bed. After resin preparation, sample was processed: 100 µL of serum 100% was diluted in 750 µL of binding buffer and divided into 10 columns prepared before. Each column was quickly mixed to obtain a suspension and placed into a microcentrifuge tube. These tubes were next incubated for 10 min at RT on an orbital shaker and centrifugated at 10,140 rpm for 1 min. Filtrate was recovered and reapplied to resin bed. A new incubation for 10 min at RT on an orbital shaker was achieved, and after a centrifugation at 10,140 rpm for 1 min, filtrate was collected, pooled and stored at -20°C before use. The filtrate solution was evaporated to dryness using a Savant SpeedVac integrated vacuum concentrator (SPD 1030-230 system, Thermo Scientific) for 2 hours and then reconstituted with distilled water to its original volume. The serum albumin level, measured to verify the effectiveness of the procedure, was found to be negligible (120 mg/L). Aptamer working solution was prepared as previously, and depleted serum was diluted to obtain final fraction from 10% to 75%. Aptamer and depleted serum working solutions were finally mixed into individuals wells.

Target-dependent response of probes in human serum samples
Aptamer working solution was made as previously. Serum working solution was prepared at the intended fraction from 100% serum solution. Successive dilutions of L-Tym were made from 0 to 300 µM. Each dilution was next mixed with aptamer working solution in new tubes to form the aptamer-target complex, and incubated at RT for 5 min in the dark. Complex and serum working solution were then mixed into individual wells. This protocol was employed for L-Tym titration curve at different final serum fraction, from 1% to 90%. For L-Tym-binding curve without serum, serum working solution was replaced by binding buffer. Similar experiments were carried out for other aptamer systems, using target titration at 50% of final serum fraction. Control experiments using mutated aptamer probes and non-cognate ligands were performed following the same protocol.

Target-dependent response of probes in serum samples supplemented with exogenous serum albumin
After preparation of aptamer working solution, successive dilutions of L-Tym were achieved. Each dilution was mixed with a working solution containing HSA and serum (HSA final concentration: 500 µM, final serum fraction: 1%). Aptamer and L-Tym/HSA/serum working solutions were then mixed into individual wells.

Duplexed aptamer sensor
CS and aptamer working solutions were prepared as above mentioned. Serum working solution was prepared at the intended fraction from 100% serum solution. Successive dilutions of L-Tym were made from 0 to 1,800-3,600 µM, depending on the final matrix fraction, due to analyte solubility constraints in the stock solution. Each dilution was then mixed with aptamer working solution in new tubes to form complex aptamer-target and incubated at RT for 5 min. CS working solution was then added in each tube. Aptamer/target/CS and serum working solutions were finally mixed into individual wells. This experimental procedure was employed for serum final fraction ranging from 1 to 10% in FA-based signaling mode and from 5 to 90% in FI-based signaling mode. As previously, serum working solution was replaced by binding buffer for L-Tym or D-Tym titration curve in the absence of serum.

Standard addition method

L-Tym system: *S*amples containing 50% pooled serum (commercial and lab-prepared from multiple subjects), spiked with different L-Tym concentrations (X_{theor} from 0.4 µM to 2 µM), were first prepared and aliquoted. Different known amounts of L-Tym were then added to the aliquots to obtain 0.5 µM, 1 µM, 2 µM, 4 µM (for X_{theor} = 0.4 µM), 1 µM, 2 µM, 4 µM, 8 µM (for X_{theor} = 0.7-1.5 µM) and 1 µM, 2 µM, 5 µM, 8 µM (for X_{theor} = 2.0 µM) final concentrations in serum samples. A target concentration of 300 µM was also used to achieve saturated binding and thus obtain the minimum FA value. The maximum FA value was defined using a non-spiked sample.

Theo system: samples containing 50% serum from randomly selected individuals (not treated with the drug) were first spiked with different clinically relevant concentrations of Theo, i. e. X_{theor} from 12.5 µM to 62.5 µM, respectively. They were aliquoted and different known amounts of Theo were subsequently added to the resulting solutions at final concentrations of 5 µM, 25 µM, 75 µM, 500 µM. A target concentration of 2000 µM was also applied to an aliquot in order to obtain the minimum FA value corresponding to binding saturation. The maximum FA value was determined from a spiked sample using the mutated probe 5'-TR-apt$_{mut}$-Theo instead of 5'-TR-apt-Theo.

In both cases, the oligonucleotide working solution, prepared as before, and target/serum working solution were finally mixed into individual wells.

Quantification of total protein, albumin and γ-globulins in human serum

The protein analysis methods we used are those that are routinely implemented at the Grenoble University Hospital and, as such, are fully validated for clinical application. Their modalities are briefly described hereafter. Total protein was measured using the Weichselbaum Biuret method, in which cupric sulfate in an alkaline medium reacts with peptide bonds to form a purple complex. The resulting solution was read for absorbance at 545 nm. Albumin was quantified either by electrophoresis (see below) or by an immunoassay. For the latter, immunoturbidimetry was implemented, in which samples were reacted with a specific anti-albumin antiserum to generate immune complexes, and turbidity was measured at 340/700 nm. The concentrations of γ-globulins and albumin were measured by means of a fully automated capillary electrophoresis system. In this method, serum proteins were separated in free solution within silica capillaries using a pH 9.9 buffer, then detected directly by absorbance at 200 nm. The electropherograms obtained were then used to measure the major protein fractions and calculate their respective concentrations based on the total protein content.

Table S1: Representative overview of homogeneous fluorescent aptamer sensors for small-molecule analytes when directly confronted with serum specimens, i.e. in the absence of any prior clean-up procedure. The serum fraction on which the work was based is given as it appears in the literature. However, the exact sample dilution procedure is not systematically available, and there is a disparity in the expression of the dilution factor, some taking into account dilution in the final solution analyzed, others not. Consequently, the data below indicates some matrix fractions that are overestimated in relation to what was actually present in the final reaction medium. These are indicated in brackets where we were able to estimate it from the data provided. As biofluid content increases, disturbances become increasingly evident (highlighted in blue), regardless of the sensor design or target type. With respect to the latter, when they significantly associate with serum proteins (indicated in italics), working directly in the biological environment may result to a response related to their non-protein-bound fraction,[S13-S15] possibly contributing to the performance changes observed during the transposition from the buffer to the highly loaded samples.

LOD: limit of detection; $K_{1/2}$: target concentration needed to obtain 50% of the absolute signal gain; NP: nanoparticle; TWJ: three-way junction; SSB: single-stranded DNA binding protein; GO: graphene oxide.

**Small-molecule targets**	**Sensor design**	**Serum fraction**	**Performance changes (*vs* clean buffer)**
ATP<br>Anal. Chem. **2011**, 83, 5122-5127	Duplexed aptamer	0.5%	No significant change
Phenylalanine<br>Nat. Chem. **2014**, 6, 1003-1008	Duplexed aptamer	0.5%	No significant change
Dabigatran etexilate<br>Talanta **2020**, 218, 121132	Duplexed aptamer	0.5%	No significant change
Cocaine<br>Anal. Chem. **2012**, 84, 3554−3560	Enzymatic protection	1%	No significant change
Adenosine, dopamine, *17β-estradiol*<br>Biosens. Bioelectron. **2016**, 79, 198-204	Quencher	1%	No significant change
Arginine-vasopressin<br>Anal. Methods, **2016**, 8, 2383	Intrinsic conformational change	1%	No significant change
Adenosine<br>Chem. Sci. **2020**, 11, 2735	Fluorescent dye	1%	No significant change
Serotonin<br>Anal. Chem. **2023**, 95, 9076-9082	Light-up aptamer	1%	No significant change
Serotonin<br>Talanta **2024**, 277, 126363	Fluorescent dye	1%	No significant change
Digoxin<br>Microchem. J. **2024**, 200, 110452	Duplexed aptamer and amplification	1%	No significant change
Lactate<br>Sens. Actuators B **2025**, 425, 137015	Sandwich-like	1%	No significant change
Arginine-vasopressin<br>Talanta **2025**, 293, 128102	Split aptamer	1%	No significant change
Adenosine<br>Anal. Chem. **2015**, 87, 3139-3143	Split aptamer	2%	No significant change
Digoxin<br>Talanta **2020**, 219, 121235	NP	2%	No significant change
Vancomycin<br>Small **2025**, 21, 2407799	Fluorescent dye	2%	No significant change
Tetrodotoxin<br>Spectrochim. Acta A **2019**, 219, 411-418	Fluorescent dye	2.5%	No significant change
17β-Estradiol, cocaine<br>Sens. Actuators B **2020**, 324, 128586	Split aptamer	10% (→ 2.5%)	No significant change
Tobramycin<br>Microchem. J. **2021**, 160, 105657	TWJ destabilization	2.5%	~15-fold increase in LOD
Tyrosinamide<br>Sens. Actuators B **2025**, 437, 137725	SSB	2.5%	No significant change
ATP<br>ACS Sens. **2025**, 10, 246-253	Light-up aptamer	3%	No significant change
Ampicillin<br>Biosens. Bioelectron. **2020**, 164, 112329	Duplexed aptamer	3.3%	No significant change
Adenosine<br>Analyst **2017**, 142, 2247	Modified split aptamer	10% (→ < 5%)	No significant change
ATP<br>Biosens. Bioelectron. **2015**, 70, 188-193	Intrinsic conformational change	Up to 5%	Up to 2-fold increase in LOD in 5% serum
Cocaine<br>Anal. Bioanal. Chem. **2017**, 409, 3993-4000	Intrinsic conformational change	Up to 5%	~6-fold increase in $K_{1/2}$ (estimated from Fig. S3) and 4-fold increase in LOD in 5% serum
Theophylline<br>Microchim. Acta **2019**, 186, 494	GO and amplification	5%	5-fold decrease in linear range
ATP<br>Sens. Actuators B **2020**, 323, 128653	Duplexed aptamer and amplification	5%	No significant change
ATP<br>Anal. Chem. **2013**, 85, 11518-11523	Duplexed aptamer and amplification	10%	No significant change

Tryptophan<br>Talanta **2015**, 131, 672-677	GO	10%	Assay response suppressed
ATP<br>Sens. Actuators B **2018**, 273, 70-75	Duplexed aptamer and amplification	10%	No significant change
Theophylline<br>RSC Adv. **2019**, 9, 33898	NP	10%	No significant change
ATP<br>Analyst **2020**, 145, 434	Duplexed aptamer and amplification	10%	No significant change
Piperaquine<br>Sci. Transl. Med. **2021**, 13, eabe1535	Duplexed aptamer	10%	~10-fold increase in $K_{1/2}$ (estimated from Fig. 2 and 3)
ATP<br>Nat. Commun. **2021**, 12, 4654	Strand invasion	10%	No significant change
Theophylline<br>ACS Chem. Biol. **2022**, 17, 2121-2129	Duplexed aptamer	10%	~30% loss in signal gain (estimated from Fig. 5 and 6)
ATP<br>Sensors **2022**, 22, 2425	GO and amplification	10%	No significant change
Adenosine<br>J. Am. Chem. Soc. **2023**, 145, 7540-7547	Duplexed aptamer	10%	~35% loss in signal gain (estimated from Fig. 4)
Citrate<br>Adv. Sensor Res. **2024**, 3, 2300167	Duplexed aptamer	10%	Assay response suppressed
17β-Estradiol<br>Anal. Methods **2025**, 17, 3220	Fluorescent dye	10%	No significant change
Lactate<br>Sens. Actuators B **2025**, 442, 138075	Fluorescent dye	10%	~10-fold decrease in signal gain (estimated from Fig. 3 and S17)
Kanamycin<br>Analysis & Sensing **2025**, 5, e202400099	Duplexed aptamer	10%	~70% loss in signal gain (estimated from Fig. 7 and S8) and 3-fold increase in LOD
Tobramycin<br>Biosens. Bioelectron. **2026**, 303, 118569	Fluorescent dye	10%	No significant change
Rapamycin<br>Anal. Chem. **2026**, 98, 4508-4517	Duplexed aptamer	Up to 10%	Up to ~20-fold increase in LOD in 10% serum
Adenosine<br>Anal. Chem. **2012**, 84, 7203-7211	SSB	Up to 20%	20% increase in background noise in 20% serum
ATP<br>Anal. Chem. **2013**, 85, 1424-1430	GO	20%	~10-fold increase in LOD*
Arginine-vasopressin<br>Anal. Chim. Acta **2018**, 1001, 143e150	Kissing aptamer	Up to 20%	Assay response suppressed from 10% serum
Quinine, cocaine, cholic acid<br>ACS Appl. Bio Mater. **2021**, 4, 6732-6741	Fluorescent dye	Up to 20%	Up to ~40% loss in signal gain and ~5-fold increase in LOD in 20% serum
Cocaine<br>Anal. Chem. **2014**, 86, 11100-11106	Fluorescent dye	Up to 25%	~20-fold decrease in signal gain in 25% serum (estimated from Fig. S8)
Flunixin<br>J. Am. Chem. Soc. **2023**, 145, 12407-12422	Duplexed aptamer	40%	> 25-fold increase in $K_{1/2}$ (estimated from Fig. S16) and 100-fold increase in LOD
25-hydroxyvitamin D3<br>Luminescence **2012**, 27, 51-58	Intrinsic conformational change	Up to 50%	Inversion of the assay response from 10% serum
Cocaine<br>J. Am. Chem. Soc. **2024**, 146, 3230-3240	Duplexed aptamer	50%	~10-fold increase in $K_{1/2}$ (estimated from Fig. 3)
Lactate<br>Angew. Chem. Int. Ed. **2023**, 62, e202212879	Duplexed aptamer	Up to 90%	~6-fold decrease in signal-to-noise ratio in 90%serum (estimated from Fig. S10)
Vancomycin<br>Sens. Actuators B **2025**, 436, 137681	Duplexed aptamer	90%	~5-fold increase in $K_{1/2}$ (estimated from Fig. 4 and 5) and LOD
Methotrexate<br>ACS Sens. **2021**, 6, 2436-2441	Duplexed aptamer	Up to 95%	~30-fold increase in LOD in 95% serum
Adenosine<br>Anal. Chem. **2012**, 84, 7852-7856	Duplexed aptamer	~100%	Assay response suppressed**

*Experimental conditions in the serum are somewhat different from those in the clean buffer (8 *vs* 10 μg/mL GO)
**The loss of response occurs when steady-state analysis is implemented. A significant target-dependent signal is nevertheless obtained through time-resolved measurements; however, in this case, it is not possible to perform a comparison with clean buffer conditions, as such data are not available.

Table S2. Z' value for the different sensors in pooled-serum-rich samples (F = 25-90%). This statistical metric provides practical insights into the reliability and quality of an assay, considering both its dynamic range and experimental variability.[S10] A value ≥ 0.5 indicates excellent discrimination between background noise and output signal.[S10] The Z' values obtained with all the sensors developed fall within the range of those found in quantitative analysis.[S16,S17]

**Sensors**	L-Tym				Coc	Ade	Theo	L-Trp	Dpm	Mtx
**F**	25%	50%	75%	90%	50%	50%	50%	50%	50%	50%
**Z' value**	0.88	0.85	0.73	0.83	0.88	0.70	0.73	0.77	0.87	0.76

Table S3. List of the serum specimens from individuals (pat id: 1-9) used for investigating the L-Tym sensor under variable matrix conditions (see Figure 5a). In particular, these were selected so that the concentrations of total proteins, albumin, and γ-globulins (mainly consisting of immunoglobulins) covered a very wide range (the extreme values are underlined in gray). The colored dots refer to the experimental curves reported in Figure 5a.

Samples	[Protein] g/L	[Albumin] g/L	[γ-globulin] g/L
pat id 1	96	30.4	51.0
pat id 2	63	43.3	NA
pat id 3	60	36.2	6.3
pat id 4	81	36.4	16.7
pat id 5	54	25.1	6.2
pat id 6	70	26.5	17.3
pat id 7	72	44.9	9.0
pat id 8	48	19.2	6.5
pat id 9	72	45.0	9.1

Normal concentration range:
[Protein] = 60-83 g/L; [Albumin] = 35-50 g/L; [γ-globulin] = 7-16 g/L

Table S4. Recovery rates at F = 50% for the L-Tym sensor, measured using spiked pooled sera from different commercial batches or prepared in-house (pat id: 10-13), and for the Theo sensor, measured using spiked sera from randomly selected individuals (pat id: 14-23). Theo, a bronchodilator used to treat asthma and chronic obstructive pulmonary disease, has a narrow therapeutic range (15-55 µM in 50% serum).[S18] Accordingly, the samples were supplemented with the drug at concentrations spanning this therapeutic window. Both assays demonstrated overall performance comparable to, or better than, that of a number of heterogeneous-phase systems tested under similar conditions, as shown in Table S5.

	**Applied concentration X_{theor} (µM)**	**Estimated concentration U (µM)**	**Recovery rate % ($100*U/X_{theor}$)**
**Pooled sera (L-Tym)**			
Batch 0000303263	0.40	0.316	79.00
In-house batch	0.70	0.965	137.86
Batch 0000303263	0.80	0.722	90.25
Batch 0000399415	0.80	1.012	126.50
Batch 0000298223	1.00	0.860	86.00
In-house batch	1.30	1.230	94.62
Batch 0000298223	1.50	1.278	85.20
Batch 0000298223	1.50	0.999	66.60
Batch 0000303263	2.00	2.126	106.30
Batch 0000303263	2.00	2.054	102.70
			Overall mean: 97.5 ± 21.6
**Individual sera (Theo)**			
pat id 14	12.50	9.807	78.46
pat id 15	12.50	10.052	80.42
pat id 16	18.75	12.591	67.15
pat id 17	18.75	16.040	85.55
pat id 18	31.25	19.477	62.33
pat id 19	31.25	18.623	59.59
pat id 20	48.875	56.784	116.18
pat id 21	48.875	43.113	88.21
pat id 22	62.50	76.663	122.66
pat id 23	62.50	74.745	119.59
			Overall mean: 88.0 ± 23.7

Table S5. Recovery rates reported in the literature for aptamer-based assays performed on untreated, serum-rich samples. Only studies reporting explicit information on final serum fraction and analyte recovery rate (either directly stated or readily derivable) were included. All identified systems operated under heterogeneous-phase conditions. One study reported a negligible recovery of the thrombin analyte (Table 2 of reference [S19]). Among the remaining studies, one used a signal-amplifying sandwich assay to detect TNF-α in 25% serum specimens from individual donors (Table 1 of reference [S20]). Other studies employed electrochemical (E-AB) sensors exposed to pooled serum at levels between 50% and 100% (Table 2 of reference [S22], Table S1 of reference [S23] and Table 1 of reference [S24]). Since numerical values were not explicitly provided in references [S21] and [S25], they were obtained by digitizing data from Figures S8, S14, and Figure 5A of these studies, respectively, using the open-source tool WebPlotDigitizer (see Figure S7 for an example of extraction process). As such, the overall recovery values presented for these E-AB sensors are approximate.

**Target**	**Assay**	**Sample**	**Overall recovery rate %**	**Ref.**
Thrombin	Sandwich-type ICP-MS assay	100% pooled human serum	5-25 fM range:<br>none	[S19]
TNF-α	Sandwich-type fluorescent assay	25% human serum from individuals	6.25-800 fM range:<br>104.3 ± 20.2	[S20]
Cocaine	E-AB sensor	100% pooled bovine serum	8-800 μM range:<br>120 ± 40, 105 ± 20 and 75 ± 5, depending on sensor fabrication batches	[S21]
Doxorubicin	E-AB sensor	100% pooled bovine serum	2-40 μM range:<br>105 ± 10, 95 ± 15 and 75 ± 15, depending on sensor fabrication batches	[S21]
Alpha-fetoprotein	E-AB sensor	50% pooled human serum	1-10 mg/L range:<br>112.1 ± 8.5	[S22]
Carbamazepine	E-AB sensor	100% pooled human serum	0.1-10 μM range:<br>92.2 ± 16.2<br>0.015-5 μM range:<br>98.9 ± 12.0	[S23]
Serotonin	E-AB sensor	50% pooled human serum	0.1-100 nM range:<br>99.2 ± 3.3	[S24]
Vancomycin	E-AB sensor	100% pooled human serum	2-10 μM range:<br>110 ± 70	[S25]

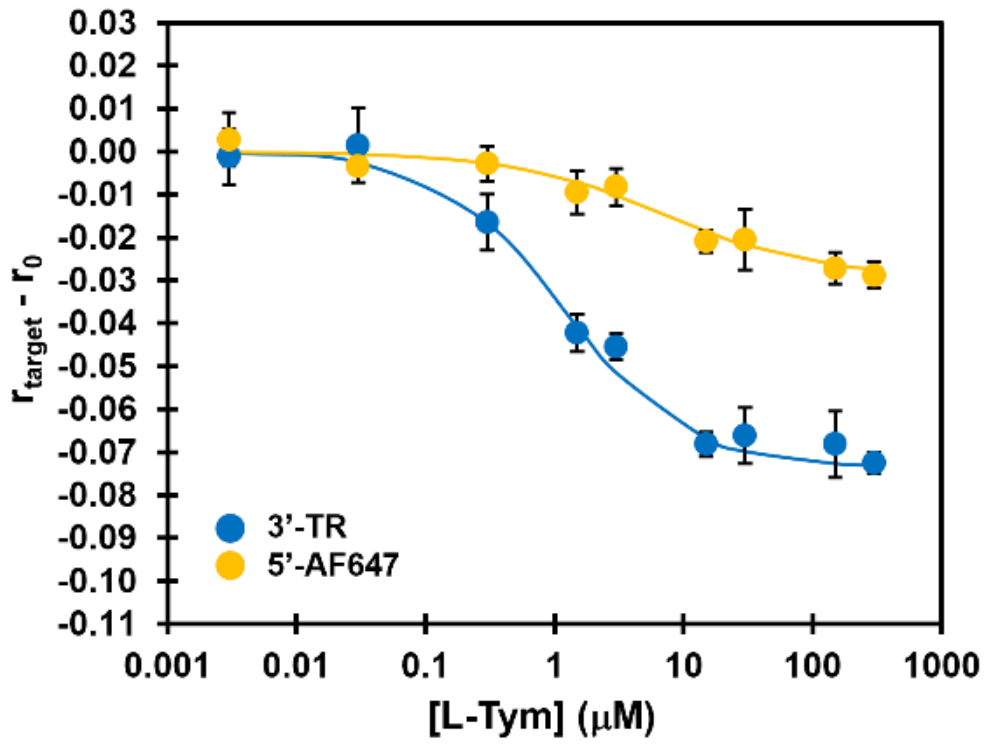

Figure S1. L-Tym-dependent FA response curve using 5'-AF647-apt-Tym in pooled serum (F = 50%). For comparison, the target-binding curve using 3'-TR-apt-Tym in the same conditions (shown in Figure 2c) is also depicted. r_{target} is FA with increasing target and r_0 is FA without target. Error bars represent SD from three independent experiments. The weaker signal gain observed with the AF647 conjugate may be attributed to a shorter excited-state lifetime (1 ns *vs* 4 ns for TR), which caused a higher baseline FA value in the buffer (0.220 *vs* 0.150 for TR conjugate) and, consequently, a smaller FA change upon exposure to matrix proteins. In addition, the attached AF647 dye could modify the probe's ability to adsorb onto matrix proteins[S26] and/or interact with the target,[S27] both of which could contribute to the poorer apparent affinity ($K_{1/2}$ ~7 μM *vs* ~1 μM for TR conjugate).

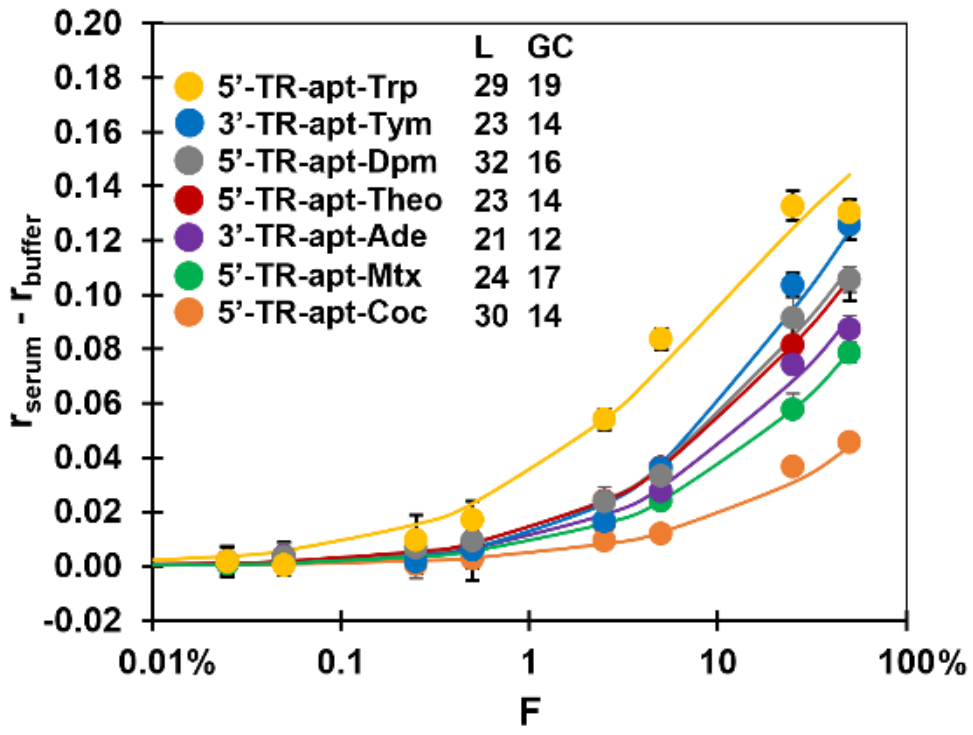

Figure S2. FA change of the different probes used in this study as a function of F (pooled serum) under target-free conditions and for a final Mg^{2+} concentration of 10 mM. r_{buffer} is FA in clean buffer and r_{serum} is FA with added biofluid. Error bars represent SD from three independent experiments. The binding curves fell within a range of serum fractions, reflecting their different affinities for matrix proteins. Sequence length (L) and GC content are recognized as parameters that modulate oligonucleotide-protein interactions.[S28,S29] Given the lack of clear correlation between these factors and FA variations, it is likely that the structural state of the free probe, specifically, its degree of unfolding, also contributes to the observed binding behavior. In this regard, it is worth noting that apt-Coc, which is known to adopt a partially structured three-way junction in its unbound form,[S30] exhibits the lowest affinity for matrix proteins.

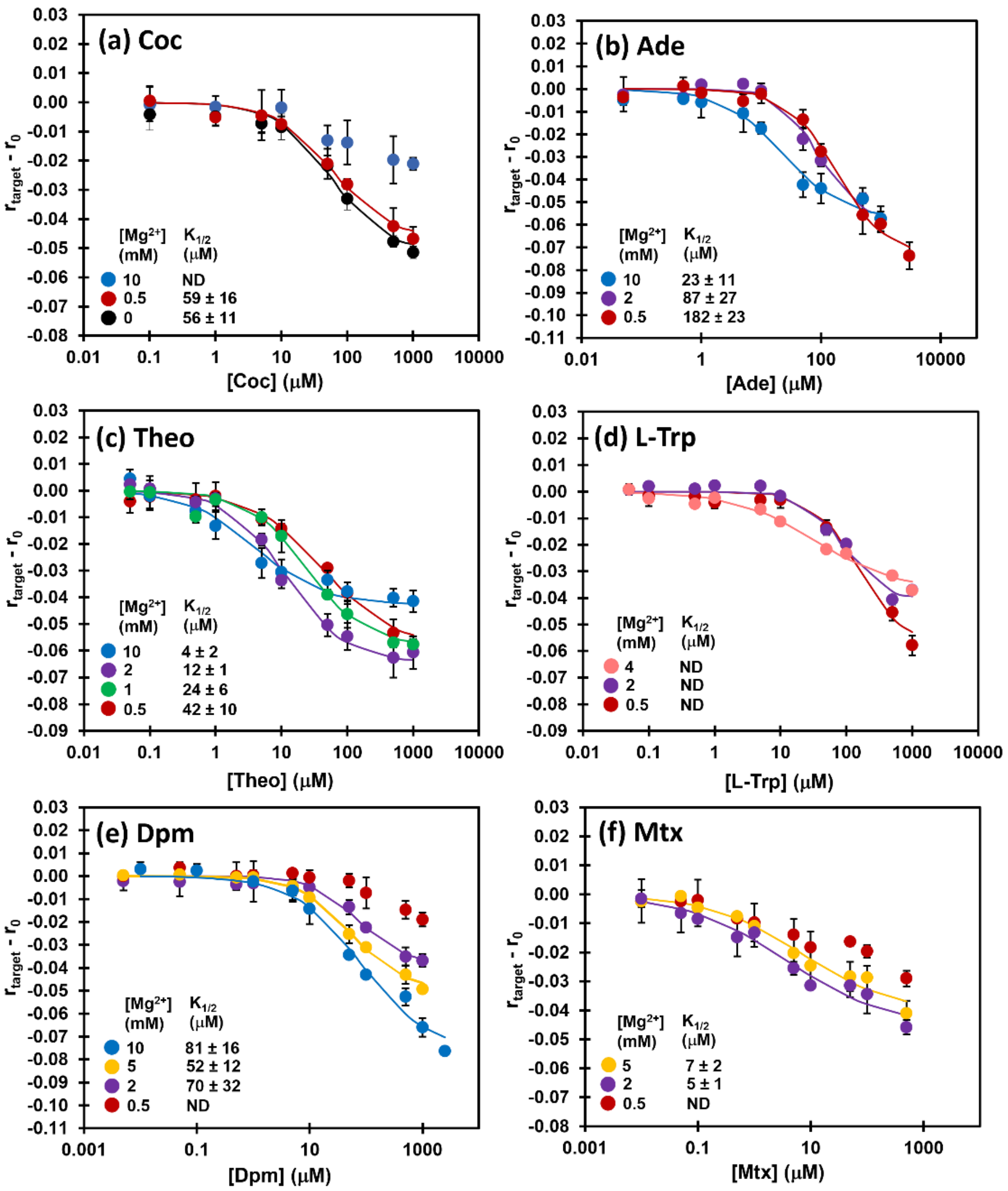

Figure S3. Target-dependent FA response curves for **(a)** Coc using 5'-TR-apt-Coc, **(b)** Ade using 3'-TR-apt-Ade, **(c)** Theo using 5'-TR-apt-Theo, **(d)** L-Trp using 5'-TR-apt-Trp ($K_{1/2}$ was not determined, as L-Trp is 20-100 μM in blood[S4]), **(e)** Dpm using 5'-TR-apt-Dpm and **(f)** Mtx using 5'-TR-apt-Mtx in 50% pooled serum at various final Mg^{2+} concentrations. r_{target} is FA with increasing target and r_0 is FA without target. Error bars represent SD from three independent experiments. It was observed that variation in the concentration of the divalent cation affects both the signal gain and the apparent affinity of the sensors, with the overall behavior depending on the aptamer considered. This is likely because Mg^{2+} may act at multiple levels of the signaling mechanism. In particular, it is assumed to modulate binding between the target-free probe and matrix proteins by affecting (i) its folding-unfolding dynamics[S31] and (ii) electrostatic interactions between the DNA backbone and cationic protein residues.[S32] Modification of the inactive, protein-bound state of the aptamer could therefore alter both the signal amplitude and the apparent affinity.[S33] The cation concentration may also affect the proper folding of the aptamer in its target-bound state,[S31] thereby further leading to Mg^{2+}-dependent variations in the response.

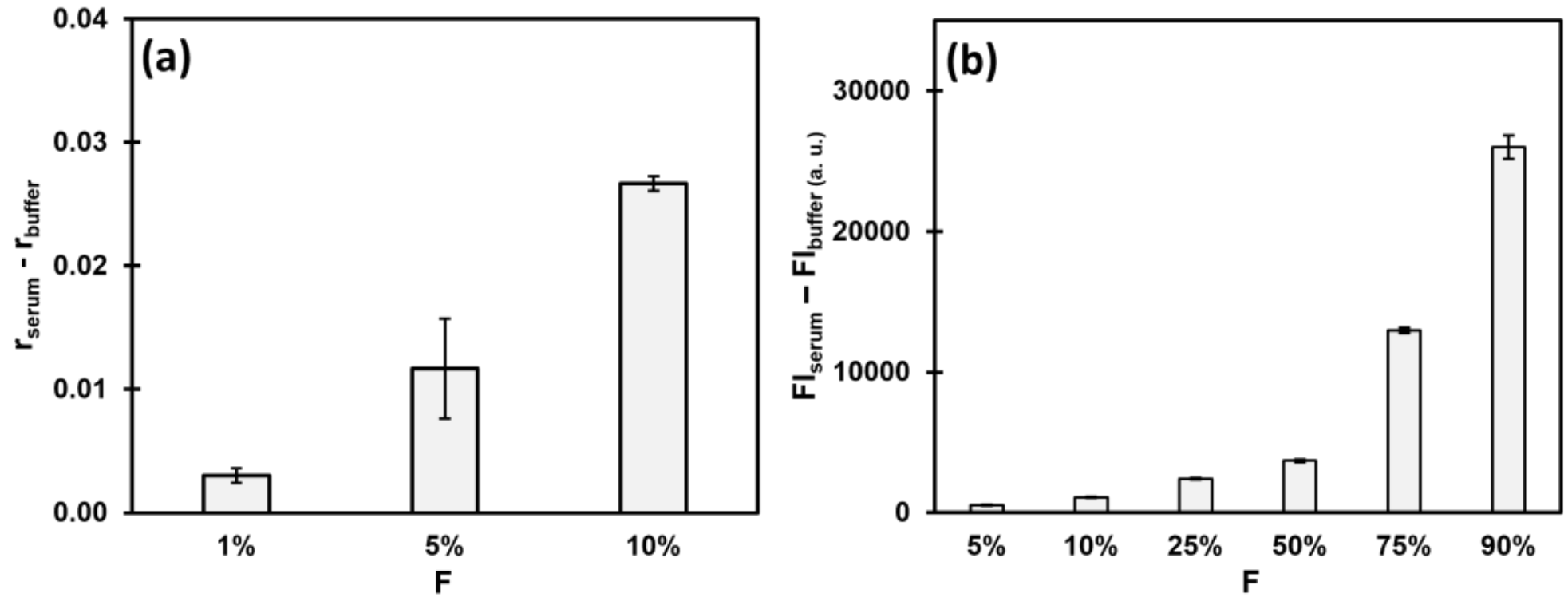

Figure S4. **(a)** FA change for the 5'-TR-CS-Tym:apt-Tym hybrid (1:1 stoichiometry) when incubated with pooled serum (F = 1-10%) in the absence of L-Tym. r_{buffer} is FA in clean buffer and r_{serum} is FA with added biofluid. The increase in FA with rising F reflected the occurrence of non-specific binding of matrix proteins within the duplexed aptamer sensor framework. **(b)** FI change for the 3'-TR-CS-Tym:5'-BHQ2-apt-Tym hybrid (1:1 stoichiometry) when incubated with pooled serum (F = 5-90%) in the absence of L-Tym. FI_{buffer} is FI in clean buffer and FI_{serum} is FI with added biofluid. Error bars represent SD from three independent experiments.

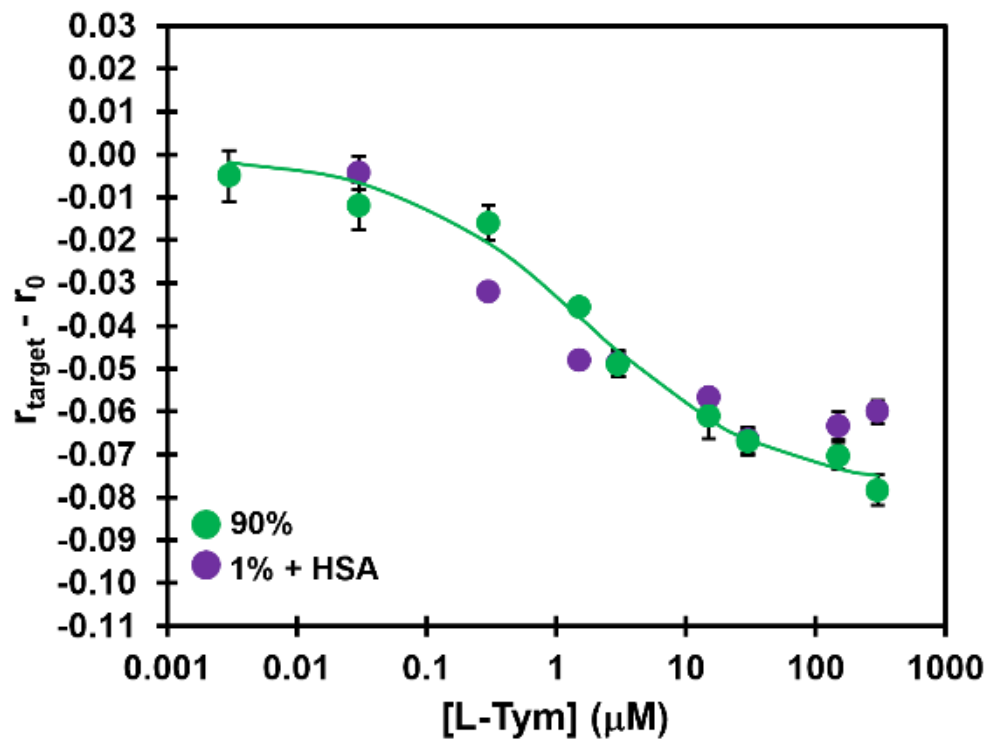

Figure S5. L-Tym-dependent FA response curve using 3'-TR-apt-Tym in highly diluted pooled serum (F = 1%) supplemented with 500 μM exogenous albumin (HSA). r_{target} is FA with increasing target and r_0 is FA without target. Error bars represent SD from three independent experiments. The target-dependent response was similar to that of the 90%-matrix sensor used as a reference, indicating that the system could be readily adapted to detect analytes when their initial concentrations in serum-rich samples exceed the test's dynamic range.

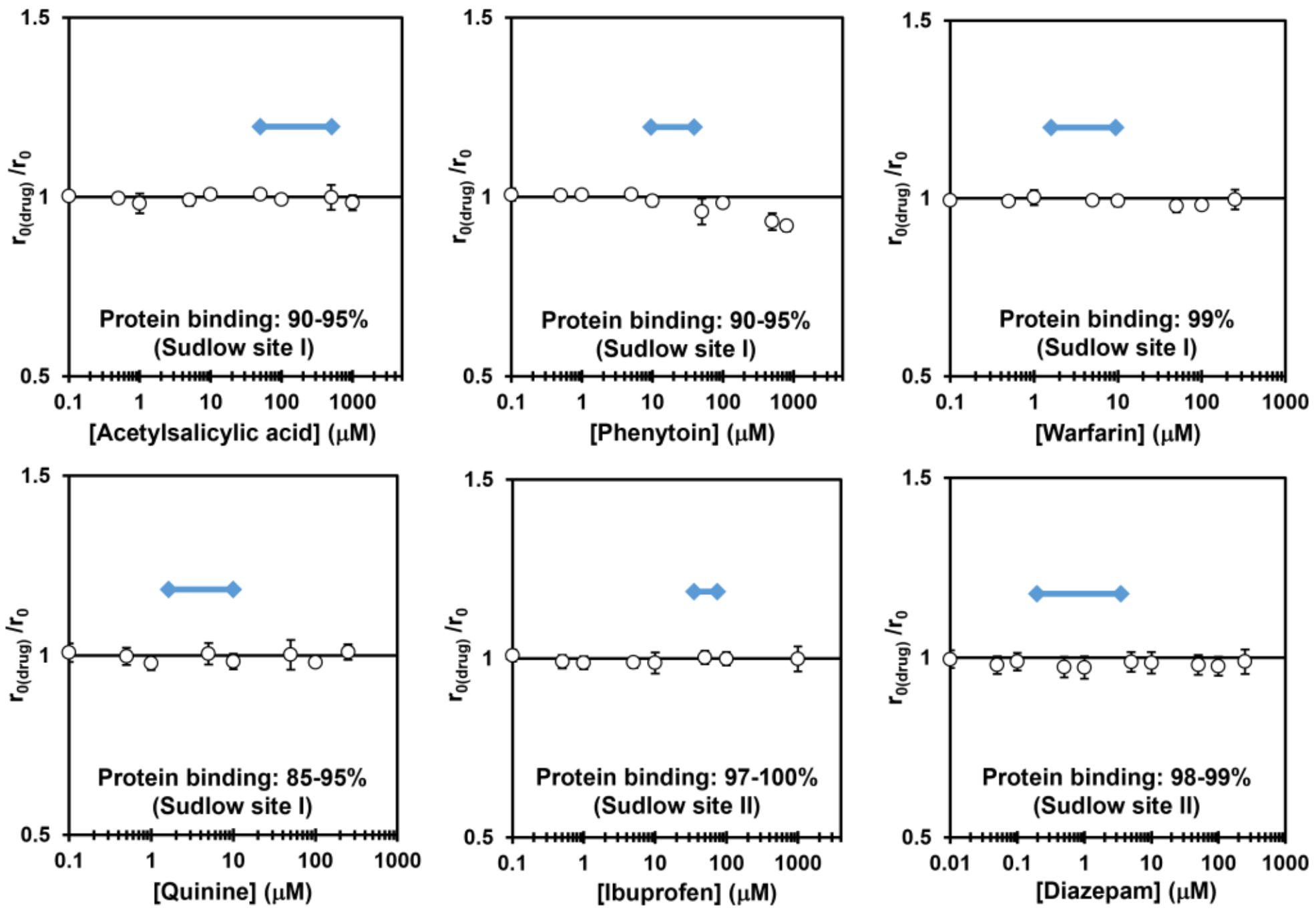

Figure S6. Effects of increasing concentrations of some representative drugs added to pooled serum (F = 50%) on the normalized FA signal of 3'-TR-apt-Tym in the absence of target. Their protein-binding properties, including the primary binding site for albumin, and therapeutic blood concentration range[S18] (in blue), are presented for each drug. $r_{0(drug)}$ is FA with drug present and r_0 is FA without drug. Error bars represent SD from three independent experiments. The lack of appreciable changes in FA for the tested compounds suggests that no interference occurs between the drugs and the probe for binding to matrix proteins, at least within therapeutic blood concentration ranges.

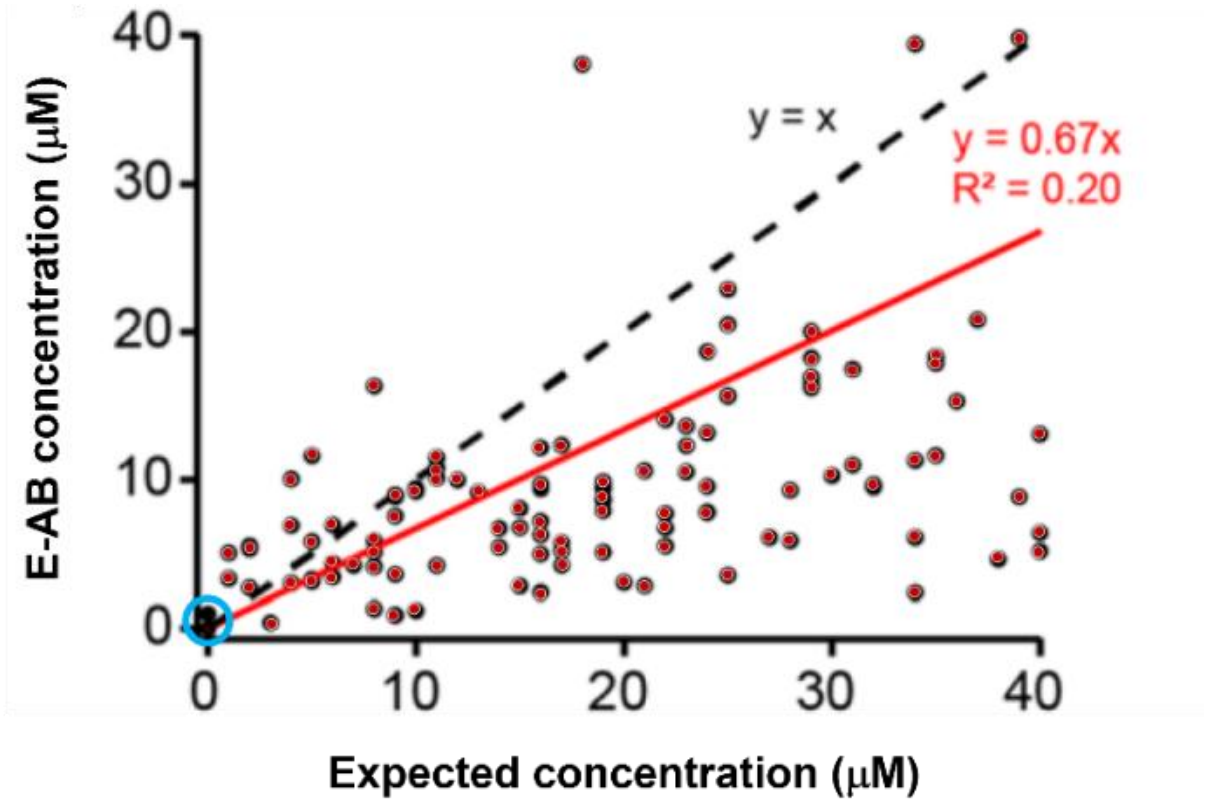

Figure S7. Example of numerical data extraction using the open-source software WebPlotDigitizer (https://automeris.io/). The plot shows the concentration measured by the E-AB sensor (corresponding to U) as a function of the expected concentration (corresponding to X_{theor}) of vancomycin in whole pooled serum (adapted from Figure 5A of reference [S25] under the Creative Commons CC BY-NC 3.0 Licence). Each point could be manually selected (in red) after prior calibration of the X,Y axes according to their concentration range. The X and Y values were then extracted, and the recovery values were calculated. Note that we excluded spike concentrations too close to zero (blue circle). The overall recovery rate was approximately 70 ± 65% when all the data points are considered. In the most accurate 2-10 μM range of vancomycin,[S25] it was around 110 ± 70%.

References

[S1] L. Challier, R. Miranda-Castro, B. Barbe, C. Fave, B. Limoges, E. Peyrin, C. Ravelet, E. Fiore, P. Labbé, L. Coche-Guérente, E. Ennifar, G. Bec, P. Dumas, F. Mavré, V. Noël, *Anal. Chem*. **2016**, *88*, 11963-11971.
[S2] S. Perrier, C. Ravelet, V. Guieu, J. Fize, B. Roy, C. Perigaud, E. Peyrin, *Biosens. Bioelectron.* **2010**, *25*, 1652-1657.
[S3] M-D. Nguyen, M. T. Osborne, G. T. Prevot, Z. R. Churcher, P. E. Johnson, L. Simine, P. Dauphin-Ducharme, *Biosens. Bioelectron.* **2024**, *265*, 116680.
[S4] Y, Wu, S. Ranallo, E. del Grosso, A. Chamaro-Garcia, H. L. Hennis, N. Milosavic, K. Yang, T. Kippin, F. Ricci, M. Stojanovic, K. W. Plaxco, *Bioconjugate Chem.* **2023**, *34*, 124-132.
[S5] Y. A. Kaiyum, E. H. P. Chao, L Dhar, A. A. Shoara, M-D. Nguyen, C. D. Mackereth, P. Dauphin-Ducharme, P. E. Johnson, *ChemBioChem* **2024**, *25*, e202400493.
[S6] Z. Zhang, O. Oni, J. Liu, *Nucleic Acids Res.* **2017,** *45,* 7593–7601.
[S7] X. Lin, Y. Huang, J. Huang, H. Yuan, Y. Luo, Z. Lu, Y. Ao, J. Huang, S-B. Chen, Z. Miao, L. Huang, *Angew. Chem. Int. Ed.* **2025**, *64*, e202504107.
[S8] E. H. P. Chao, E. Largy, Y. A. Kaiyum, M-D. Nguyen, B. Vialet, P. Dauphin-Ducharme, P. E. Johnson, C. D. Mackereth, *bioRxiv preprint* **2025**, https://doi.org/10.1101/2025.11.27.690961.
[S9] J. He, J. Wang, M. Zhang, G. Shi, *ACS Sens*. **2021**, *6*, 2436-2441.
[S10] D. M. Jameson, J. A. Ross, *Chem. Rev.* **2010,** *110,* 2685-2708.
[S11] S. Pang, S. Cowen, *Sci. Rep.* **2017**, *7*, 17542.
[S12] M. Conrad, P. Fechner, G. Proll, G. Gauglitz, *Biosensors* **2023**, *13*, 849.
[S13] B. Madru, F. Chapuis-Huguon, E. Peyrin, V. Pichon, *Anal. Chem.* **2009,** *81,* 7081-7086.
[S14] O. Alkhamis, J. Canoura, C. Willis, L. Wang, J. Perry, Y. Xiao, *J. Am. Chem. Soc.* **2023**, 145, 12407−12422.
[S15] Y. Liu, J. O. Mack, M. Shojaee, A. Shaver, A. George, W. Clarke, N. Patel, N. Arroyo-Curras, *ACS Sens.* **2024**, *9*, 228−235.
[S16] H. Harma, G. Sarrail, *J.* Kirjavainen, *E.* Martikkala, I. Hemmila, P. Hanninen, *Anal. Chem.* **2010**, *82,* 892-897.
[S17] M. Dominguez, I. Moreno, A. Torano, *J. Immunol. Methods* **2019**, *474*, 112645.
[S18] M. Schulz, S. Iwersen-Bergmann, H. Andresen, A. Schmoldt, *Crit. Care* **2012**, *16*, R136.
[S19] Q. Zhao, X. Lu, C-G. Yuan, X-F. li, X. C. Le, *Anal. Chem.* **2009,** *81,* 7484-7489.
[S20] D. Wu, E. Katilius, E. Olivas, M. Dumont Milutinovic, D. R. Walt, *Anal. Chem*. **2016**, *88*, 8385-8389
[S21] H. Li, P. Dauphin-Ducharme, G. Ortega, K. W. Plaxco, *J. Am. Chem. Soc.* **2017**, 139, 11207-11213.
[S22] W. Li, M. Chen, J. Liang, C. Lu, M. Zhang, F. Hu, Z. Zhou, G. Li, *Anal. Methods*, **2020**, *12*, 4956.
[S23] S. Chung, N. K. Singh, V. K. Gribkoff, D. A. Hall, *ACS Omega* **2022**, *7*, 39097−39106.
[S24] Z. Hu, R. Zhu, G. Figueroa-Miranda, L. Zhu, L. Feng, A. Offenhausser, D. Mayer, *Biosensors* **2023**, *13*, 881.
[S25] J. Mack, R. Murray, K. Lynch, N. Arroyo-Curras, *Sens. Diagn*. **2024**, *3*, 1533-1541.
[S26] D. Roncancio, H. Yu, X. Xu, S. Wu, R. Liu, J. Debord, X. Lou, Y. Xiao, *Anal. Chem*. **2014**, *86*, 11100-11106.
[S27] A. Guitton Auberty, E. Peyrin, J. Nenadovic, J-L, Ravanat, S. Perrier, *Sens. Actuators B-Chem.* **2025**, *437*, 137725.
[S28] G. A. Nevinsky, *Int. J. Mol. Sci.* **2021**, *22*,1369.
[S29] N. Wu, M. Zandieh, T. Yang, J. Liu, *Anal. Chem*. **2023**, *95*, 8948-8955.
[S30] Z. R. Churcher, D. Garaev, H. N. Hunter, P. E. Johnson, *Biophys. J*. **2020**, *119*, 1147-1156.
[S31] C. Kaiser, M. Vogel, B. Appel, J. Weigand, S. Muller, B. Suess, J. Wachtveitl, *J. Mol. Biol.* **2023**, *435*, 168253.
[S32] H. Ausserwöger, *G.* Krainer, T. J. Welsh, N. Thorsteinson, E. de Csilléry, T. Sneideris, M. M. Schneider, T. Egebjerg, G. Invernizzi, T. W. Herling, N. Lorenzen, T. P. J. Knowles, *Proc. Natl. Acad. Sci. USA* **2023***, 120,* e2210332120.
[S33] S. G. Harroun, C. Prévost-Tremblay, D. Lauzon, A. Desrosiers, X. Wang, L. Pedro, A. Vallée-Bélisle, *Nanoscale* **2018**, *10*, 4607-4641.

Author Contributions

A. Barbaroux conducted the experiments on aptamer sensors, processed and analyzed the corresponding data, and participated in writing the manuscript. E. Fiore and M. Zoubeidi carried out the preliminary experiments implementing the in-situ aptamer sensor strategy. S. Vezirian and M. Henry helped conduct the additional experiments and analyses related to the revision process. B. Chovelon supervised the protein analyses on individual samples. M. C. DeRosa secured the necessary funding, supervised the initial experiments conducted on this topic, and reviewed the data and the manuscript. E. Peyrin, B. Chovelon and C. Ravelet conceptualized the study, obtained the necessary research funding, designed the experiments, analyzed the data, and wrote the manuscript. E. Peyrin was responsible for project administration.
